# *PbDELLA*-*PbMYB56*-*PbCYP78A6* module regulates GA_**4 + 7**_-induced pseudo-embryo development and parthenocarpy in pear (*Pyrus bretschneideri*)

**DOI:** 10.1093/hr/uhaf021

**Published:** 2025-01-21

**Authors:** Haiqi Zhang, Jingjing Cheng, Xue Wang, Pingyuan Dai, Hongjuan Zhang, Fengli Zhou, Chengquan Yang, Rui Zhai, Zhigang Wang, Lingfei Xu

**Affiliations:** College of Horticulture, Northwest A&F University, Taicheng Road No. 3, Yangling, Shaanxi Province, 712100 China; Key Laboratory of Horticultural Crop Germplasm Innovation and Utilization (Co-construction by Ministry and Province), Institute of Horticulture, Anhui Academy of Agricultural Sciences, Nongke South Road No. 40, Luyang District, Hefei, Anhui Province, 230001 China; College of Horticulture, Northwest A&F University, Taicheng Road No. 3, Yangling, Shaanxi Province, 712100 China; College of Horticulture, Northwest A&F University, Taicheng Road No. 3, Yangling, Shaanxi Province, 712100 China; College of Horticulture, Northwest A&F University, Taicheng Road No. 3, Yangling, Shaanxi Province, 712100 China; College of Horticulture, Northwest A&F University, Taicheng Road No. 3, Yangling, Shaanxi Province, 712100 China; College of Horticulture, Northwest A&F University, Taicheng Road No. 3, Yangling, Shaanxi Province, 712100 China; College of Horticulture, Northwest A&F University, Taicheng Road No. 3, Yangling, Shaanxi Province, 712100 China; College of Horticulture, Northwest A&F University, Taicheng Road No. 3, Yangling, Shaanxi Province, 712100 China; College of Horticulture, Northwest A&F University, Taicheng Road No. 3, Yangling, Shaanxi Province, 712100 China; College of Horticulture, Northwest A&F University, Taicheng Road No. 3, Yangling, Shaanxi Province, 712100 China

## Abstract

Parthenocarpy can ensure fruit setting without fertilization and generate seedless fruits. *PbCYP78A6* has been shown to play a role in gibberellin (GA)-induced parthenocarpy in pears. However, the transcriptional response mechanism of *PbCYP78A6* to GA remains unclear. In this study, using a yeast one-hybrid assay combined with co-expression analysis, *PbMYB56* was initially identified as a transcription regulator of *PbCYP78A6*, which was further confirmed by electrophoretic mobility shift assay (EMSA) and dual-luciferase reporter assays. The biofunction of *PbMYB56* was further verified using transient transgene tests, stable transgenic pear callus and tomato. *PbMYB56* overexpression resulted in reduced cell death and higher fluorescence intensity after fluoresce diacetate (FDA) staining, as well as delayed fruit-drop by increasing *PbCYP78A6* expression in unpollinated pear fruitlets and callus. In contrast, silencing *PbMYB56* caused cell death and early fruit-drop with decreased *PbCYP78A6* expression. Moreover, after emasculation, heterologous overexpression of *PbMYB56* induced parthenocarpy and enlarged seed size in pollinated tomato fruits. Silencing *SlMYB56*, a homolog of *PbMYB56* in tomatoes, resulted in smaller fruit and seed size, and these traits were restored by co-overexpression with *PbCYP78A6*. Furthermore, we investigated the protein interaction between PbMYB56 and PbDELLA, which is crucial component of the GA signaling pathway. This interaction inhibited PbMYB56-induced transcriptional activation of *PbCYP78A6*. Co-overexpression of *PbMYB56* and *PbDELLA* contributed to reduced seed development and loss of parthenocarpy potential in tomatoes. Collectively, our study identifies PbDELLA-PbMYB56-*PbCYP78A6* as a regulatory module of GA_4 + 7_-induced pseudo-embryo and parthenocarpy development, offering insights into the mechanism underlying parthenocarpy formation in pears.

## Introduction

Parthenocarpy is characterized by fruit formation without fertilization, making it a vital method for producing seedless fruits. For horticultural crops where fruits are the primary consumable, seedlessness is a desirable trait that appeals to consumers and enhances ease of consumption. The production of parthenocarpic fruits has been applied to various species, including tomatoes [1], watermelon [2], grapes [3], citrus [4], and pears [5]. Identifying parthenocarpy-associated genes provides genetic resources and a theoretical basis for producing and utilizing seedless varieties.

The role of GA is central to understanding the parthenocarpic fruit-setting mechanism. Auxins and GA are often considered signals from ovules following successful pollination and fertilization [6–8]. Artificially synthesized auxins and GA can successfully mimic these signals, enabling fruit development independent of fertilization [1, 9]. Auxins induce fruit setting partially through the synthesis of GA [1, 6]. Manipulating GA biosynthesis or signaling pathways can decouple fruit setting from fertilization [10, 11]. GA cascades boost central metabolic enzyme activity, increasing sink capacities for fruit setting [12]. DELLA protein plays a pivotal role in GA-induced fruit set [11–13], although the downstream components interacting with DELLA in GA-mediated fruit set remain unclear.

GA induces *AtCYP78A9* and *AtCYP78A6* to act on the integument, with overexpression of either resulting in the formation of large, seedless siliques [14, 15]. Similarly, *PbCYP78A6* operates downstream of GA to promote cell proliferation and parthenocarpy in pears [16]. Genes influencing ovule integument are strongly associated with parthenocarpy. Reprogramming of ovule integument has been shown to enhance the parthenocarpic potential of tomato *agamous-like 6* [17]. Additionally, overexpression of *SlKLUH*, a cytochrome P450 (CYP) cell proliferation factor, stimulates integument growth in unfertilized ovules, leading to parthenocarpy [17]. These findings indicate that signals regulating integument development play a vital role in fruit setting, as genes governing this process may be critical for fruit development independent of fertilization. Beyond their fundamental role as key hormones in fruit set and development, GAs have also been shown to positively influence seed size in crops, such as rice and soybean [18, 19]. However, the mechanisms linking GA, ovule development, and parthenocarpy remain poorly understood.

Applying parthenocarpy to pears (*Pyrus bretschneideri*) helps overcome the challenges of self-incompatibility in fruit setting, thereby improving productivity. Exogenous hormones, particularly auxins, GA, and cytokinins, are commonly used to induce parthenocarpy in pears [5, 20–23]. Among these, histological, hormonal, and transcriptomic analyses have shown that GA_4 + 7_ can successfully decouple fruit set from pollination and fertilization [22]. Despite the discovery of genes associated with parthenocarpy, only a few have been conclusively identified and validated as causal factors. In a previous study, *PbCYP78A6* played a pivotal role in cell proliferation associated with the parthenocarpic formation in pears [16]. Thus, *PbCYP78A6* emerges as an essential target for further research aimed at elucidating the mechanisms of parthenocarpy, offering new insights into the processes governing fruit set and development.

*PbCYP78A6*, acting downstream of gibberellins, promotes cell proliferation and parthenocarpy in pears [16]. However, the mechanism by which GA induces *PbCYP78A6* and the involvement of a potential transcription factor mediating communication between GA and *PbCYP78A6* remains unknown. In this study, the *PbMYB56*-*PbCYP78A6* pathway, functioning downstream of GA_4 + 7_, is identified as a significant molecular pathway potentially mediating the interaction between pseudo-embryo formation and fruit development. PbMYB56 interacts with the PbDELLA protein, contributing to gibberellin signaling. Gene expression, transcription regulation, functional analyses, and protein interaction studies reveal the potential role of PbMYB56 in pseudo-embryo formation and pear parthenocarpic fruit set. Collectively, these findings identify and unveil a novel GA-induced pathway regulating pseudo-embryo formation and parthenocarpy in pears.

## Results

### Association of *PbMYB56* and *PbCYP78A6* expression with pseudo-embryo development in GA_4 + 7_-induced parthenocarpy

**Fig. 1:**
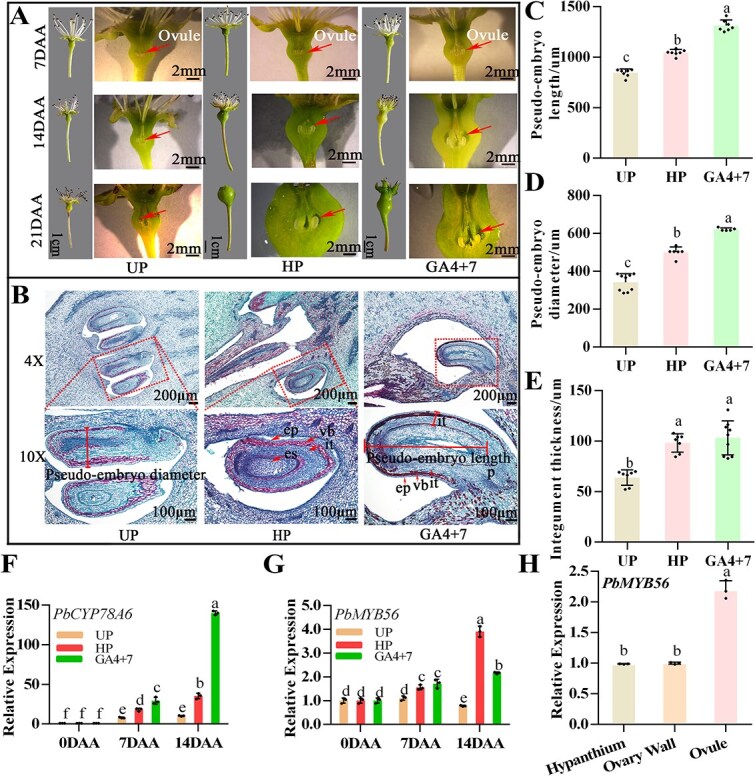
Expression levels of *PbCYP78A6* and *PbMYB56* linked to GA_4+7_-induced pseudo-embryo development and parthenocarpy. (A) Phenotypes (left) and cross-sectional observations (right) of ovaries from unpollinated (UP), hand-pollinated (HP), and GA_4 + 7_-treated groups at 7, 14, and 21 days after anthesis (DAA). Pseudo-embryos are marked by red arrows. (B) Paraffin sections showing longitudinal views of ovaries in UP, HP, and GA_4 + 7_ groups at 14 DAA. Abbreviations: es, embryo sac; ep, epidermis; vb, vascular bundle; it, integument; p, placenta. Images in the lower panel are magnified views of the areas outlined in the upper images. (C–E) Measurements of pseudo-embryo length (C), pseudo-embryo diameter (D), and integument thickness (E) in ovaries of the UP, HP, and GA_4 + 7_ groups, as indicated in (B). Three ovaries were paraffin-sectioned, and at least three pseudo-embryos were analyzed. (F–G) Relative expression levels of *PbCYP78A6* (F) and *PbMYB56* (G) in treated pear ovaries. (H) Relative expression of *PbMYB56* in pear fruit tissues, including the hypanthium, ovary wall, and ovule. Different lowercase letters (a, b, c) denote significant differences. (*P* < 0.05) based on one-way analysis of variance. Data are derived from three biological replicates and represent means with standard deviations.

In parthenocarpy induction, ovaries exhibited significant inflation in the hand-pollination (HP) and GA_4 + 7_ treatment groups, with notable ovary cavity expansion, especially in the GA_4 + 7_ group, compared to the unpollinated (UP) treatment group at 14 DAA (Fig. 1A, Supplemental Fig. S1A).

From 7 to 21 DAA, UP ovaries ceased growing, while those of the HP and GA_4 + 7_ groups continued to expand (Fig. 1A). Developing seeds were observed in HP-treated ovaries, whereas a “pseudo-embryo” formed in GA_4 + 7_-treated ovaries (Fig. 1A). Paraffin-embedded longitudinal sections revealed that pseudo-embryos were significantly larger in GA_4 + 7_-treated ovaries than in the UP group (Fig. 1BD$\hbox{--}$). Additionally, pseudo-embryo length and diameter were markedly greater in GA_4 + 7_-treated ovaries compared to UP pseudo-embryos (Fig. 1C and D). The integument was thicker in HP ovules and pseudo-embryos of GA_4 + 7_-treated ovaries than in the UP group (Fig. 1E). Pseudo-seeds were detected in mature fruits of the GA_4 + 7_-treated group, while true seeds were found in HP-treated pears (Supplemental Fig. S1B).

To further investigate the mechanism underlying pseudo-embryo development and parthenocarpy in pears, we conducted a whole-genome analysis to identify *CYP78A* subfamily members and assess their expression levels under different treatments. Among the CYP78A subfamily genes tested, *PbCYP78A6* exhibited a notable and significant increase in expression in the ovary, hypanthium, and sepal of the GA_4 + 7_-treated and HP groups compared to the UP group (Supplemental Fig. S1C). Our previous study demonstrated that *PbCYP78A6* mediates cell proliferation and parthenocarpy and that its expression responds to GA-induced parthenocarpy in pears [16]. These findings confirm that *PbCYP78A6* (LOC103964254) plays a distinct role among *CYP78A* subfamily members in pears, tightly associating with pseudo-embryo development and parthenocarpy.

Yeast one-hybrid (Y1H) library screening identified *PbMYB56* as a candidate gene acting upstream of *PbCYP78A6*. The expression of *PbMYB56* was stimulated in both fruit-set treatments (HP and GA_4 + 7_), mirroring the induced expression of *PbCYP78A6* (Fig. 1F and G). Furthermore, *PbMYB56* exhibited higher expression in the ovule compared to other fruit tissues, including the hypanthium and ovary wall (Fig. 1H). Phylogenetic analysis showed that PbMYB56 is most similar to MdMYB56 and SlMyb-like from other species, which are classified within the R2R3-MYB subgroup 21. PbMYB56 also shares homology with AtMYB56 and AtMYB69 (Supplemental Fig. S2). Sequence alignment revealed that PbMYB56 contains a conserved MYB domain (Supplemental Fig. S3A). The C-terminal and N-terminal regions of PbMYB56 exhibited transcriptional self-activation activity in a yeast two-hybrid (Y2H) assay. Yeast grew well on SD/−Trp-His-Ade selection medium and turned blue with the addition of X-α-Gal solution, whereas the R2R3 domain of PbMYB56 showed no transcriptional self-activation activity (Supplemental Fig. S3B). The 35S:PbMYB56-RFP (Red Fluorescence Protein) signals were localized to the nucleus, contrasting the ubiquitous RFP signal in the 35S:RFP control (Supplemental Fig. S3C). *PbMYB56 was* expressed substantially in the ovaries, with an increasing trend from −2 to 4 DAA (Supplemental Fig. S3D). Together, these results suggest that PbMYB56 functions as a transcription factor, and might be associated with *PbCYP78A6* expression, pseudo-embryo development, and parthenocarpy.

### PbMYB56 promotes *PbCYP78A6* expression by directly binding to its promoter

**Fig. 2:**
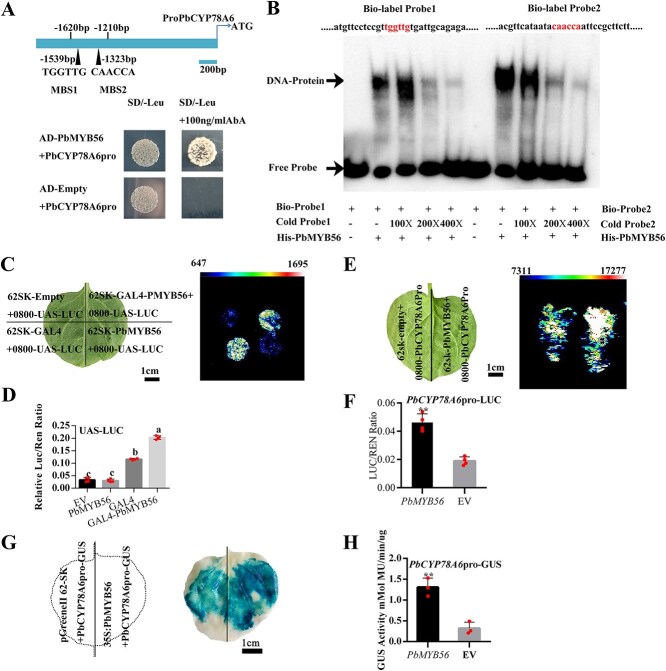
PbMYB56 enhances *PbCYP78A6* expression by promoter binding. (A) Diagram of the *PbCYP78A6* promoter and Y1H assay results. (B) Electrophoretic mobility shift assay showing PbMYB56 binding specifically to MYB-binding site (MBS) 1 and MBS 2 in the *PbCYP78A6* promoter. (C-D) PbMYB56 functions as a transcriptional activator. (C) Configuration of cotransformed vectors in tobacco leaves and fluorescence intensity indicating firefly luciferase activity via plant live imaging. (D) Quantification of the firefly luciferase (LUC)/*Renilla* (REN) ratio from (C). The LUC gene, driven by the 5XUAS (GAL4 upstream activating sequence) promoter, served as the reporter, while the CaMV 35S promoter-driven *REN luciferase* gene was the internal reference. *PbMYB56* was fused to GAL4, acting as the effector. (E-F) PbMYB56 activates *PbCYP78A6* transcription. (E) Configuration of cotransformed vectors in tobacco leaves and fluorescence intensity indicating firefly LUC activity via plant live imaging. (F) Relative LUC activity measured in tobacco leaves. An empty vector was used as a negative control. (G-H) PbMYB56 activates *PbCYP78A6* transcription in a GUS enzyme activity assay. (G) Configuration of cotransformed vectors in tobacco leaves (left) and corresponding image (right). (H) GUS enzyme activity. Data represent at least three biological replicates in (D), (F), and (H). ^*^P < 0.05.

To elucidate the relationship between *PbMYB56* and *PbCYP78A6*, we examined the impact of PbMYB56 on *PbCYP78A6* transcription. The Y1H assay demonstrated that PbMYB56 binds to *PbCYP78A6* promoter at two MYB-binding sites (MBSs) (Fig. 2A).

We synthesized promoter segments of *PbCYP78A6* containing two MYB-binding sites (MBSs): (TGGTTG and CAACCA) and conducted EMSA. A distinct shift was observed when PbMYB56 was incubated with biotin-labeled probes, indicating binding, whereas no shift occurred without the PbMYB56 protein. The shift diminished upon coincubation with an excess of unlabeled probes (Fig. 2B). Additionally, the DNA$\hbox{--}$protein complex shifts disappeared when using probes with mutations in both MBSs (Supplemental Fig. S4). To assess the transcriptional activation potential of PbMYB56, we fused it to GAL4 and observed a significant increase in fluorescence intensity corresponding to firefly LUC activity. This was quantitatively supported by the LUC/*Renilla* (REN) fluorescence ratio (Fig. 2C and D; Supplemental Fig. S5A). Further luciferase assays demonstrated that PbMYB56 directly activates *PbCYP78A6* expression (Fig. 2E–F; Supplemental Fig. S5B). Histochemical analysis revealed that co-overexpression of *PbMYB56* resulted in more intense blue staining of GUS compared to the control, and enhanced GUS enzyme activity confirmed the transcriptional activation of *PbCYP78A6* by PbMYB56 through promoter binding (Fig. 2G and H; Supplemental Fig. S5C). Collectively, these findings demonstrate that PbMYB56 binds to MBSs in the *PbCYP78A6* promoter to stimulate its expression.

### *PbMYB56* stimulates cell activity and proliferation with enhanced *PbCYP78A6* expression in pear fruitlets and calli

**Fig. 3:**
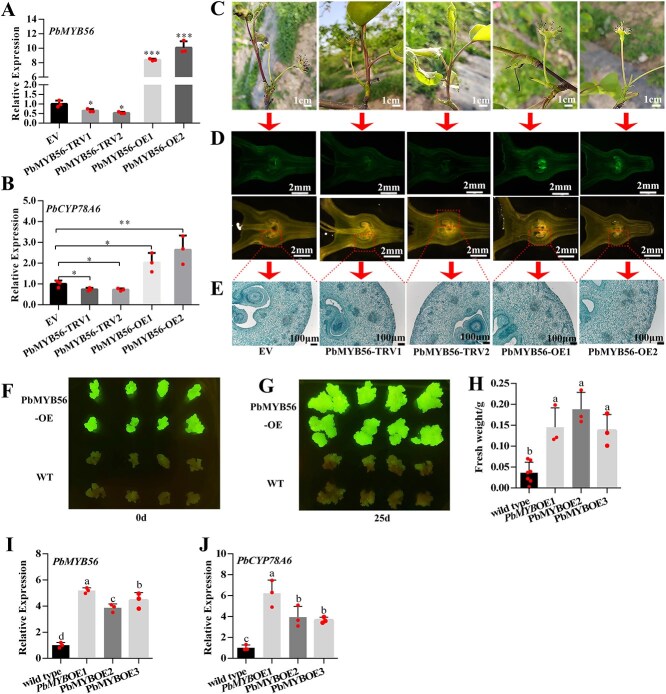
*PbMYB56* enhances cell activity and proliferation by upregulating *PbCYP78A6* expression in pear fruitlets and callus. (A) Relative expression levels of *PbMYB56* in transient transgenic pear fruitlets: *PbMYB56*-silenced lines 1 and 2 (*PbMYB56*-TRV1/2) and *PbMYB56*-overexpression lines 1 and 2 (*PbMYB56*-OE1/2), compared to those infected with the empty vector (EV). (B) Relative expression levels of *PbCYP78A6* in *PbMYB56*-TRV1/2 and *PbMYB56*-OE1/2 transient transgenic pear fruitlets. (C) Representative phenotypes of *PbMYB56*-TRV1/2 and *PbMYB56*-OE1/2 transient transgenic pear fruitlets observed at 14 days after treatment (DAT). Each treatment included 10 infiltrated pear inflorescences. (D) Detection of green fluorescence in *PbMYB56*-TRV1/2 and *PbMYB56*-OE1/2 transient transgenic pear fruitlets at 7 DAT using fluorescein diacetate (FDA) staining. The red dotted box indicates the area selected for paraffin sectioning. (E) Histological examination of paraffin sections from *PbMYB56*-TRV1/2 and *PbMYB56*-OE1/2 transient transgenic fruitlets. Three fruitlets were used for paraffin sectioning. (F) Identification of transgenic pear calli based on green fluorescence protein (GFP) signals. (G) Transgenic pear calli overexpressing *PbMYB56* after 25 days of cultivation. (H) Changes in the fresh weight of pear calli cocultured for 25 days. (I) Expression levels of *PbMYB56* in transgenic and wild-type (WT) pear calli. (J) Expression levels of *PbCYP78A6* in transgenic and WT pear calli. Different lowercase letters (a, b, c) indicate significant differences (*P* < 0.05), determined by one-way analysis of variance. Data are based on three biological replicates and are represented as means with standard deviations.

To examine the role of *PbMYB56* in parthenocarpic fruit set, we developed a transient transgenic system in pear inflorescences in *vivo* (Supplemental Fig. S6A). Using *PbCYP78A6* promoter-driven GUS gene vector, pollinated fruitlets stained overexpressing the construct blue, whereas those overexpressing the empty vector showed minimal staining (Supplemental Fig. S6B), which confirming the efficacy of the transgenic process. Through this system, we obtained *PbMYB56*-overexpressing and silenced transgenic pear fruitlets. *PbMYB56* expression increased approximately eightfold in overexpressing fruitlets and decreased twofold in silenced fruitlets compared to the control (Fig. 3A).

Correspondingly, *PbCYP78A6* expression increased in *PbMYB56*-overexpressing fruitlets and decreased in *PbMYB56*-silenced fruitlets (Fig. 3B). At 14 days after treatment (DAT), *PbMYB56* overexpressing fruitlets remained green and exhibited high fruit setting rates (Fig. 3C and Supplemental Fig. S6C), while *PbMYB56*-silenced fruitlets turned yellow and experienced fruit-drop, similar to the empty vector control (Fig. 3C; Supplemental Fig. S6C, D). fluorescein diacetate (FDA) staining revealed weak green fluorescence in *PbMYB56*-silenced ovaries at 7 DAT, whereas strong green fluorescence was detected in P*bMYB56*-overexpressing ovaries, particularly in the ovule (Fig. 3D; Supplemental Fig. S6E). Histological examination showed that *PbMYB56* overexpression increased hypanthium thickness and cell layers compared to the control, whereas silencing *PbMYB56* had no significant effect on these phenotypes (Fig. 3E; Supplemental Fig. S7A–C).

We developed a stable transgenic pear callus line overexpressing *PbMYB56*. Coculturing the transgenic callus with WT callus revealed that *PbMYB56* overexpression enhanced callus proliferation (Fig. 3F and G). The biomass of the transgenic callus increased significantly (Fig. 3H), accompanied by elevated *PbMYB56* expression (Fig. 3I). Concurrently, *PbCYP78A6* transcription levels were markedly upregulated (Fig. 3J). These findings indicate that *PbMYB56* positively influences fruit development by stimulating *PbCYP78A6* expression.

### Ectopic overexpression of *PbMYB56* contributes to parthenocarpy in tomato

**Fig. 4:**
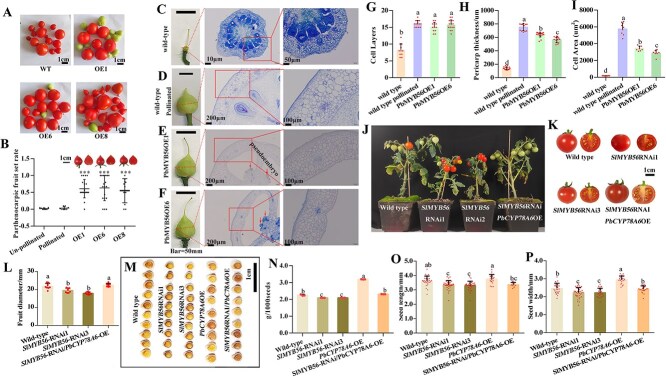
Functional characterization of *PbMYB56* in parthenocarpic induction, fruit development, and seed size in tomato. (A) Phenotypes of transgenic fruits from *PbMYB56*-overexpression lines. (B) Ectopic overexpression of *PbMYB56* leads to parthenocarpic fruit set. Ten transgenic plants were emasculated at anthesis to assess parthenocarpic capacity. Data represent three biological replicates, each containing at least three emasculated inflorescences. ^*^P < 0.05. (C–F) Phenotypes and paraffin-embedded transverse sections of ovaries in unpollinated wild-type (C), pollinated wild-type (D), and unpollinated *PbMYB56-*overexpression (OE) lines 1(E) and 6 (F). Dotted lines indicate section positions. Left images show 20× magnification of views on the right. Red arrow indicates the pseudo-embryos. (G–H) Quantification of cell layers (G), pericarp thickness (H), and cell area (I) in the pericarps of unpollinated wild-type, pollinated wild-type, and unpollinated transgenic tomato ovaries. (J) Morphological comparison of wild-type (WT), *SlMYB56-*RNAi, *PbCYP78A6*-overexpression (OE), and *SlMYB56-*RNAi/*PbCYP78A6-*OE lines. (K) Fruit phenotypes harvested from the transgenic lines in (J). (L) Fruit diameter measurements from transgenic lines. (M) Seed phenotypes from WT, *SlMYB56-*RNAi, and *SlMYB56-*RNAi/*PbCYP78A6*-OE tomato lines. (N) Quantification of seed weight in (M). (O) Length of seeds shown in (M). (P) Width of seeds shown in (M). Data represent the mean (± SD) of three biological replicates (n > 30 in N, O, and P). Different lowercase letters (a, b, c, d) indicate statistically significant differences (*P* < 0.05), determined by one-way analysis of variance. Data represent means (± SD) of three biological.

To further elucidate the function of *PbMYB56* in parthenocarpy, we generated stable transgenic tomato lines overexpressing *PbMYB56* (*PbMYB56*-OE), confirmed at both DNA and RNA levels (Supplemental Fig. S8A and B). Three *PbMYB56-*OE lines were selected for further analysis, showing no significant differences in floral morphogenesis compared to WT control plants (Supplemental Fig. S8C and D). Mature fruits from *PbMYB56*-OE lines exhibited distinct phenotypes, with substantial enhancement of fruit tips and increased fruit numbers compared to WT (Fig. 4A; Supplemental Fig. S8E).

Under pollinated conditions, *PbMYB56* overexpression significantly increased tomato seed size, seed weight, and seed length compared to WT lines (Supplemental Fig. S8F–H). When emasculated, *PbMYB56*-OE lines exhibited approximately 60% fruit set, producing seedless fruits with characteristic pointed tips, whereas WT lines did not produce fruits (Fig. 4B). In the HP group, WT lines produced seeded fruits, with no seedless fruits observed (Fig. 4B). Ovary samples were collected at 8 DAA when noticeable ovary growth was evident. Post-emasculation, WT ovaries failed to grow (Fig. 4C), while pollinated WT ovaries exhibited rapid growth (Fig. 4D). In contrast, emasculated *PbMYB56*-OE ovaries continued to grow (Fig. 4E and F). Histological analysis of fertilized WT ovaries showed significant growth in the pericarp and ovules compared to the UP group. Emasculated *PbMYB56*-OE ovaries displayed substantial pericarp expansion and the presence of a pseudo-embryo (Fig. 4CF$\hbox{--}$). Analysis revealed that *PbMYB56*-OE emasculated ovaries and pollinated WT ovaries had similar pericarp cell layer numbers, both significantly higher than UP ovaries (Fig. 4G). Quantitative assessment showed that *PbMYB56* overexpression significantly increased pericarp thickness, which was lower in UP ovaries than in pollinated ovaries (Fig. 4H). The average cell area in the pericarp of *PbMYB56*-OE emasculated ovaries was much larger than in UP ovaries (Fig. 4I). These findings indicate that *PbMYB56* overexpression contributes to parthenocarpy in tomato.

### Overexpression of *PbCYP78A6* restores the reduced fruit and seed size caused by *SlMYB56* silencing

Considering the analogous roles of *AtMYB56* and *PbMYB56* in regulating seed size, we investigated whether *SlMYB56*, a close homolog of *PbMYB56*, similarly influences seed size in tomatoes. Stable transgenic lines with *SlMYB56* silencing were generated via RNA interference (RNAi) (Supplemental Fig. S9A). Silencing *SlMYB56* resulted in delayed growth and reduced fruit production (Fig. 4J). Co-overexpression of *PbCYP78A6* in *SlMYB56*-silenced plants restored growth and fruit set to levels comparable with WT (Fig. 4K; Supplemental Fig. S9B and C). *SlMYB56*-silenced plants produced fewer and smaller fruits with reduced diameters compared to WT; this phenotype was normalized by co-overexpression of *PbCYP78A6* (Fig. 4K and L; Supplemental Fig. S9D). Additionally,*SlMYB56* silencing resulted in smaller seeds, across weight, length, and width metrics, all of which were restored to WT levels by *PbCYP78A6* co-overexpression (Fig. 4MP$\hbox{--}$). These results show that *PbCYP78A6* overexpression compensates for the reduced fruit and seed size caused by *SlMYB56* RNAi, suggesting that *PbCYP78A6* functions downstream of *SlMYB56* in promoting fruit and seed size. This evidence implies that *PbMYB56* operates upstream of *PbCYP78A6* in this regulatory pathway.

### PbDELLA directly targets PbMYB56 involved in gibberellin signaling

**Fig. 5:**
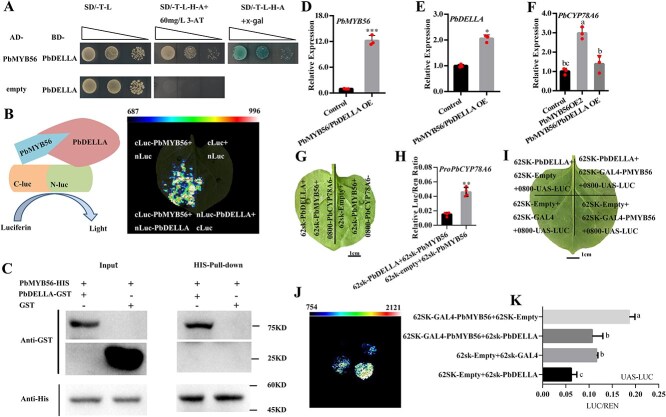
Interaction of PbDELLA with PbMYB56 reduces its transcriptional activity on *PbCYP78A6*. (A) Yeast-two-hybrid assay showing PbMYB56s’ interactions with PbDELLA. (B) Luciferase (LUC) complementation visualization demonstrating PbMYB56-PbDELLA interaction. (C) Pull-down assays confirming the interaction. Purified GST or PbDELLA-GST was incubated with PbMYB56-His with anti-His conjugated magnetic beads. Anti-His antibodies assessed pull-down efficiency by detecting PbMYB56-His, and anti-GST antibodies confirmed protein interactions by detecting PbDELLA-GST. (D) Expression of *PbMYB56* in *PbMYB56*/*PbDELLA-*OE ovaries. (E) Expression of *PbDELLA* in *PbMYB56*/*PbDELLA*-OE ovaries. (F) *PbCYP78A6* expression in *PbMYB56*/*PbDELLA*-OE and *PbMYB56*-OE ovaries. (G) Transformation mode of recombined vectors cotransformed into tobacco leaves. (H) LUC/REN ratio in (G). (I) Transformation mode of recombined vectors cotransformed into tobacco leaves. (J) Fluorescence intensity displaying firefly LUC activity via a plant live imaging. (K) LUC/REN ratio in (J). Three biological replicates were performed and error bars represent standard deviation. Statistically significant differences are indicated by different lowercase letters (a, b, c, d). Statistical tests were conducted via one-way analysis of variance (ANOVA, ^*^P < 0.05).

DELLA proteins play a core role in GA signaling. Five DELLA proteins were identified and characterized in the pear genome based on the conserved DELLA domain and phylogenetic analysis (Supplemental Fig. S10A and B). Among these five *PbDELLA* genes, LOC103943039 expression was suppressed in the HP and GA_4 + 7_ treatment groups at 7 and 14 DAA compared to the UP groups, showing increased expression from 7 to 14 DAA (Supplemental Fig. S11A–E), which differs from the others’ expression. [based on fragments per kilobase of transcript per million fragments mapped (FPKM)] of *PbDELLA* (LOC103943039) was the highest among *PbDELLA* genes in the UP condition, as reported in a previous study (Supplemental Fig. S11F; [22]). Consequently, LOC103943039 was selected for further study, as it displayed relatively high expression in the ovule and hypanthium but low expression in the ovary wall (Supplemental Fig. S11G). *PbDELLA* (LOC103943039) thus emerged as a potential key gibberellin signaling gene regulating fruit setting. Additionally, the Y2H assay revealed that PbDELLA interacts with PbMYB56 in yeast (Fig. 5A).

Protein truncation analysis of PbDELLA in the Y2H assay (Supplemental Fig. S12A) showed that the PbDELLA C-terminal domain, including SAW, PFYRE, LHR2, VHIID, and LHR1 domains, did not interact with PbMYB56. However, the N-terminal region containing the TVHYNP structure did (Supplemental Fig. S12B). The isolated TVHYNP domain also interacted with PbMYB56 (Supplemental Fig. S12B). Thus, the Y2H assay revealed that PbDELLA interacts with PbMYB56 through the TVHYNP domain. A luciferase complementation experiment further confirmed the interaction, with co-expression of PbDELLA-nLuc and PbMYB56-cLuc in tobacco leaves producing a clear signal (Fig. 5B). The interaction was validated through a GST pull-down experiment, where GST-PbDELLA pulled down His-PbMYB56, while GST alone did not (Fig. 5C). These results confirm that PbMYB56 interacts with PbDELLA in GA signaling.

### Interaction of PbDELLA with PbMYB56 diminishes the transcriptional activation of *PbCYP78A6* induced by PbMYB56

To explore the effect of PbDELLA and PbMYB56 interaction on *PbCYP78A6* transcription, transiently transgenic fruitlets co-overexpressing *PbMYB56* and *PbDELLA* (*PbMYB56-*OE/*PbDELLA*-OE) were generated using methods outlined in Fig. 3. *PbMYB56* expression increased approximately 12-fold with co-overexpression of *PbMYB56* and *PbDELLA* (*PbMYB56-*OE/*PbDELLA*-OE) (Fig. 5D), while *PbDELLA* expression increased 2-fold under the same conditions (Fig. 5E). The *PbMYB56*-OE2 line Fig. 3A, showing a comparable level of *PbMYB56* overexpression, was used to compare *PbCYP78A6* expression with that in *PbMYB56-*OE/*PbDELLA-*OE lines. Co-overexpression diminished *PbMYB56*s' ability to activate *PbCYP78A6* expression (Fig. 5F). A dual-luciferase assay revealed that co-overexpression of *PbDELLA* and *PbMYB56* significantly reduced *PbMYB56*-indcued promoter activation of *PbCYP78A6* (Fig. 5G and H; Supplemental Fig. S13A). Furthermore, PbMYB56 fused to GAL4 enhanced the LUC/REN ratio by binding to upstream activating promoter sequences driving luciferase expression, but co-overexpression with *PbDELLA* markedly reduced luciferase expression and the corresponding LUC/REN ratio (Fig. 5IK$\hbox{--}$; Supplemental Fig. S13B). Thus, PbDELLA suppresses the transcriptional activation of *PbCYP78A6* mediated by *PbMYB56*.

### Ectopic overexpression of *PbDELLA* and *PbMYB56* blocks the seed, fruit development, and parthenocarpy attributed to *PbMYB56* overexpression

**Fig. 6:**
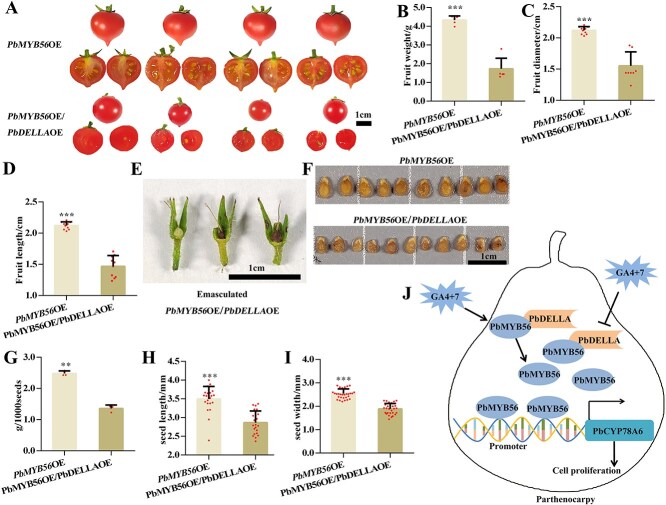
Overexpression of *PbDELLA* inhibits the function of *PbMYB56* in promoting fruit and seed development, as well as parthenocarpy in tomatoes. (A) Mature fruits produced by *PbMYB56*-overexpression (*PbMYB56*-OE) and co-overexpression of *PbMYB56* and *PbDELLA* (*PbMYB56-*OE/*PbDELLA-*OE) in tomato lines. (B–D) Measurements of fruit weight (B), diameter (C), and length (D) in *PbMYB56-*OE and *PbMYB56-*OE/*PbDELLA-*OE lines. (E) Aborted fruit set phenotypes in emasculated flowers of *PbMYB56*-OE and *PbMYB56-*OE/*PbDELLA-*O lines. (F) Seeds from fruits of *PbMYB56-*OE and *PbMYB56-*OE/*PbDELLA-*OE lines. (G–I) Evaluations of seed dry weight (G), length (H), and width (I) from seeds shown in (F). Data are presented as means ± standard deviations from three biological replicates. Statistical significance was determined using one-way analysis of variance (^*^P < 0.05). (J) A model of PbMYB56 targeted by PbDELLA to reduce the transcriptional activity of *PbCYP78A6* in GA_4 + 7_-induced parthenocarpy. Exogenous GA_4 + 7_ represses *PbDELLA* expression and activates *PbMYB56* expression, which transcriptionally upregulates *PbCYP78A6*, inducing cell proliferation and parthenocarpy formation. PbMYB56 interacts with PbDELLA, a key component of GA signaling pathway, and this interaction inhibits PbMYB56-induced transcriptional activation of *PbCYP78A6*.

To verify the impact of *PbDELLA* and *PbMYB56* interaction on *PbMYB56* function, transgenic tomato lines co-overexpressing *PbDELLA* and *PbMYB56* (*PbMYB56-*OE/*PbDELLA-*OE) were constructed (Supplemental Fig. S14A–C). Due to poor fruit set capacity in *PbMYB56-*OE/*PbDELLA-*OE lines, all co-overexpression lines were pooled for analysis. Unlike the pointed-tip shape of seeded fruits from *PbMYB56*-OE lines, *PbMYB56-*OE/*PbDELLA-*OE lines produced small, nearly round fruits with sparse seeds after natural pollination (Fig. 6A).

Fruits from *PbMYB56-*OE/*PbDELLA-*OE lines were significantly smaller than those from *PbMYB56-*OE lines, with reduced fruit weight, diameter, and length (Fig. 6B–D). After emasculation, flowers of *PbMYB56-*OE/*PbDELLA-*OE lines failed to sustain growth (Fig. 6E). Seeds from *PbMYB56-*OE/*PbDELLA-*OE fruits were significantly smaller and lighter, with narrower diameters and shorter lengths compared to seeds from *PbMYB56-*OE lines (Fig. 6F–I). Germinated seedlings from *PbMYB56-*OE/*PbDELLA-*OE lines exhibited narrower and shorter cotyledons, supported by quantitative analysis (Supplemental Fig. 14D–F). These findings highlight the restrictive role of DELLA proteins and demonstrate that co-overexpression of *PbDELLA* with *PbMYB56* blocks the effects of *PbMYB56* alone on fruit development, seed size, and parthenocarpy.

## Discussion

The mechanism underlying parthenocarpy has been extensively studied in model plants; however, research on parthenocarpy in fruit trees remains limited. This is particularly critical for pear trees, which are self-incompatible and rely heavily on successful pollination and fertilization for production. In this study, we demonstrated that PbMYB56, acting downstream of GA_4 + 7_, is directly targeted by PbDELLA, influencing *PbCYP78A6* transcription and inducing parthenogenesis. This provides a molecular framework and foundation for parthenocarpic breeding.

Previous studies applied exogenous GA to pear trees to enhance fruit set and induce parthenogenesis [5]. Although modules and key genes involved in parthenocarpy induction have been proposed [22], research has predominantly focused on the GA biosynthesis pathway and hormonal crosstalk [20, 24]. *PbCYP78A6*, identified as downstream of GA, has been implicated in seedless pear formation [16]. Due to the conserved nature of *CYP78A* genes [25–27], among all *CYP78A* subfamily members in the pear genome, only *PbCYP78A6* was transcriptionally activated by GA_4 + 7_ and pollination. Its expression pattern aligned with pseudo-ovule development in GA_4 + 7_-induced parthenocarpy. Thus, *PbCYP78A6* emerges as a key genetic factor, providing valuable insights into the mechanism of parthenocarpy.

We identified that the R2R3 transcription factor *PbMYB56*, classified in the S21 subfamily of R2R3 MYB proteins, directly activates *PbCYP78A6* by interacting with two MYB-binding sites on its promoter. Its role as a transcription factor was validated. While AtMYB56 has been reported to repress flowering by reducing *FLOWERING LOCUS T* expression [28], our findings indicate that *PbMYB56* acts as a transcriptional activator. Sequence alignment between PbMYB56 and AtMYB56 revealed amino acid differences, suggesting that *PbMYB56* possesses distinct functions in pears. Members of the S21 R2R3 MYB subfamily are known to induce cell proliferation. Specifically, *AtMYB56,* a close homolog of *PbMYB56*, promotes outer integument development and exhibits functional similarity to *AtCYP78A6*, indicating their redundant roles in pathways controlling seed size [29]. Functional analysis of *SlMYB56* and *PbMYB56* supports a conserved role of MYB56 in seed size regulation. This function aligns with that of *AtCYP78A6* [14] and *PbCYP78A6*, as identified in our study. Additionally, overexpression of *PbCYP78A6* rescued the seed phenotype caused by *SlMYB56-*RNAi, demonstrating that *MYB56* and *CYP78A6* operate within the same biological pathway. Interestingly, *PbMYB56*-overexpressing tomato plants produced fruits with pointed tips, unlike the round fruits from *PbCYP78A6*-overexpressing lines. This phenotypic difference suggests the involvement of other factors in regulating *PbCYP78A6* expression or additional target genes of *PbMYB56* that influence parthenogenesis. Although this study established the relationship between *PbMYB56* and *PbCYP78A6* during parthenogenesis in pears, further investigation is required to unravel the complex mechanisms underlying parthenocarpy.

Since woody plants have long breeding cycles, we opted to use an improved transient transgene system for this analysis. Overexpression of *PbMYB56* significantly delayed fruitlet drop-off, reduced the death signaling burst and promoted cell division in the ovaries by promoting *PbCYP78A6* expression, while silencing *PbMYB56* showed the opposite phenotype. This aligns with previous findings that members of the *CYP78A* subfamily disturb the early termination of growth by inducing cell proliferation [14,25]. Due to the transient nature of the transgene system, which only exerts effects for a limited time, we inferred the role of *PbMYB56* in pear parthenocarpy based on early fruit set phenotypes. However, we can confirm the direct transcriptional activation of *PbCYP78A6* by *PbMYB56*. This study provides the first evidence of *PbMYB56’*s role in inducing parthenocarpy in tomatoes. Interestingly, parthenocarpy produced by *PbMYB56* resulted in fruits with pointed tips, differing from the previously reported round-fruit phenotype caused by *PbCYP78A6* overexpression [16]. We speculate that variation stems from differences in the key gene mediating these effects across species. In tomatoes, *SlKLUH* has been implicated in ovule integument reprogramming and parthenocarpy formation following the loss of tomato *agamous-like 6* [17]. Furthermore, tomato CYP78A genes, including *SlCYP78A6*, a close homolog of *PbCYP78A6*, showed very low expression in WT anthesis ovules, except for *SlKLUH* [16, 17]. These findings suggest that distinct major genes regulate similar functions across species, reflecting species-specific molecular mechanisms.

DELLA proteins play a core role in GA signaling [30]. In tomatoes, silencing *DELLA* induces parthenocarpy in emasculated ovaries [13]. Based on analysis of expression and FPKM value, LOC103943039 was identified as a PbDELLA protein and a candidate target of PbMYB56. The interaction relationship was further confirmed. DELLA proteins, widely characterized for their conserved function as growth repressors, participate in various aspects of GA responses [31]. Regarding parthenocarpy, DELLA proteins have been recognized as key factors interacting with ARF/IAA and auxin signaling mediating GA-auxin interplay [11]. For example, SlARF7 and SlDELLA interact via the C-terminal portion of SlDELLA. In this study, we revealed that PbMYB56 interacts with the N-terminal domain of PbDELLA on the TVHYNP domain, offering new insight into the relationship between DELLA proteins and their target genes. While *GAMYB* acts downstream of DELLA to induce gene transcription in aleurone, the interaction appears indirect, as evidenced by a time lag between DELLA degradation and *GAMYB* transcription activation by GA [32,33]. Here, we identified PbMYB56 as a transcription factor directly targeted by PbDELLA. Co-expression with *PbDELLA* reduced the transcriptional activation of *PbMYB56*, and the expression of *PbCYP78A6*, activated by *PbMYB56,* was also diminished under these conditions. These findings establish a novel regulatory mechanism involving *PbMYB56* and *PbDELLA* in pears. A recent study reported that AtDELLA proteins play a positive role in seed size [34], conflicting with our finding that PbDELLA abolished the effects of PbMYB56 overexpression on increasing seed and fruit sizes. The potential impact of vegetative growth changes in DELLA mutants on reproductive development cannot be dismissed. For instance, silencing *SlDELLA* in tomatoes led to clustered plant growth with more leaves [13]. Additionally, *GmGA3ox1* knockout in soybean decreased seed size and stimulated the expression of photosynthesis-related genes, including those encoding ribulose-1, 5-bisphosphate carboxylase-oxygenase (Rubisco) activases [18]. The reduced seed size associated with low GA content aligns with our finding that *PbDELLA* overexpression decreased seed size. It is plausible that distinct DELLA proteins have species-specific functions, which may be particularly relevant for pear trees, where GA_4 + 7_ is more effective in inducing parthenocarpy [22], compared to GA3 in tomatoes [10]. Despite overlapping roles, different DELLA genes exhibit unique functions in repressing GA signaling in *Arabidopsis* [35]. Interestingly, seeds from *SlDELLA* mutants in tomatoes did not show a significant size increase following pollination [10]. Nevertheless, we confirmed that exogenous GA_4 + 7_ effectively induces ovule development in unpollinated ovaries, supporting its role in parthenocarpy.

The outer integument of seeds may mediate the communication between ovules and fruits. In this study, the relationship between integument and fruit development was further explored. In *Arabidopsis*, *AtMYB56* and *AtCYP78A6* enhance the outer integument layer in seed coats by increasing cell number [14,29]. *AtCYP78A6* overexpression induces large and seedless silique. In tomatoes, *SlAGL6*, primarily expressed in the immature ovule integument, plays a similar role. Loss of *SlAGL6* results in integument over-proliferation and parthenocarpy, while overexpression of its target gene *SlKLUH* stimulates integument growth in unfertilized ovules and induces parthenocarpy [17]. Those results suggest that genes regulating integument development can mimic fertilization signals, inducing fruit development and parthenocarpic fruit set. This association is supported by the presence of a “pseudo-embryo” in GA_4 + 7_-induced parthenocarpy and a “pseudo-embryo” in *PbCYP78A6-OE* or *PbMYB56-*OE emasculated parthenocarpic ovaries [16]. This evidence supports the hypothesis that the single spray of exogenous GA stimulates abnormal integument growth, which can continuously emit signals to trigger fruit setting and development, bypassing fertilization.

We propose a model for GA_4 + 7_-induced parthenocarpy in pears. Exogenous GA_4 + 7_ represses *PbDELLA* expression and stimulates *PbMYB56* expression, which activates the transcription of *PbCYP78A6*, to drive cell proliferation and parthenocarpy. PbDELLA targets PbMYB56 to inhibit its transcriptional activation of *PbCYP78A6* (Fig. 6J). These findings suggest that *PbMYB56* and *PbCYP78A6* hold significant potential for genetic engineering to promote seedless fruit production in pear breeding.

## Materials and methods

### Plant materials and experimental treatments

“Dangshansu” pear trees (*P. bretschneideri* Rehd.) and the Micro-Tom (*Solanum lycopersicum* L.) tomato variety served as plant materials for the experimental treatments. The plant materials and treatment methods were detailed in our previous study [36]. Fruitlets from each treatment group were randomly sampled at 0, 7, 14, and 21 DAA, while flowers at −2, 0, and 4 DAA were used for expression pattern analysis. All samples were immediately frozen in liquid nitrogen upon collection and stored at −80°C until analysis.

### Paraffin sectioning and data analysis

Paraffin sectioning was conducted as detailed in our previous report [16]. Longitudinal sections were used to observe the pseudo-embryo in the pear ovary. Each section's, position was standardized using the funiculus, and the pseudo-embryo length was measured as the distance from the funiculus to the top of the pseudo-embryo. The pseudo-embryo diameter was defined as the maximum distance between the upper and lower epidermis. Each treatment included replicate sections for analysis.

### RNA extraction and reverse transcription-quantitative polymerase chain reaction (RT-qPCR)

The RT-qPCR experimental procedures were conducted as described in previous reports [21]. Primer sequences are provided in Supplemental Table S1.

### Vector construction

For stable transgene expression, the PK7-203*-PbMYB56* binary vector was used as the overexpression vector. The pK7GWIWG2D*-SlMYB56* construct, generated according to our previous method [16], was used as the silencing vector. To construct the co-overexpression vector of *PbMYB56* and *PbDELLA*, the completed expression frame of the pCAMBIA1300 binary vector was fused with the PbDELLA coding sequence, cloned using specific primers. The purified PCR product was ligated into the PK7-203-*PbMYB56* binary vector linearized by Kpn I restriction endonuclease. Other recombinant vectors were prepared using the One Step Cloning Kit (ClonExpress II, Vazyme, China). Each recombinant plasmid was then transformed into *Agrobacterium tumefaciens* strain GV3101 respectively following the manufacturer’s protocol. Primers with adaptors for each vector are listed in Supplemental Table S2.

### Yeast library screening and Y1H assay

The yeast library screening and Y1H assay were conducted as previously described [21]. Briefly, the promoter region of *PbCYP78A6* (−1620 bp to −1210 bp upstream) was cloned from the pear genome and inserted into the p53-AbAi vector. The p53-pAbAi-bait vectors were linearized and transformed into Y1HGold. Integration of the plasmids into the Y1H genome was verified using Matchmaker Insert Check PCR Mix 1. Monoclonal yeast colonies were inoculated on SD/-Ura/AbA plates to determine the minimal inhibitory concentration of aureobasidin A (AbA) and establish a competent cell line. The AD-prey was then transformed into the bait yeast strains and selected on SD/−Leu plates with AD-empty vectors serving as a negative control. Positive yeast clones were used as templates to amplify DNA products for sequencing. The obtained sequences were blasted against the pyrus coding sequence database on the National Center for Biotechnology Information website to identify full target gene sequences and predict their functions. The coding sequences of target genes were cloned into pGADT7 vectors for subsequent Y1H experiments.

### Dual-luciferase assay

The 1939 bp sequence of the *PbCYP78A6* promoter upstream of the ATG start codon was cloned from the pear genome and inserted into the pGreenII 0800-LUC vector. The detailed procedures were described in our previous report [36].

### GUS staining and enzyme activity determination

The *PbCYP78A6* promoter was inserted into the pGreenII 1301 plasmid as a reporter vector using specific primers, followed by *Agrobacterium* infiltration according to the dual-luciferase procedure described previously; detailed methods are outlined in our earlier report [37]. GUS enzyme activity was measured using the GUS gene quantitative detection kit (SL7160, Coolarber, China), following the manufacturer’s protocol.

### EMSA

EMSA was performed using the Chemiluminescent EMSA Kit (GS009, Beyotime, China) according to the manufacturer's instructions. PbMYB56-His protein was obtained through prokaryotic, expression and purified using Ni-NTA Bind Resin (7sea Biotech, Shanghai, China). Bio-labeled and cold probes were synthesized by Beijing Tsingke Biotechnology Ltd. Co. Detailed procedures followed our previous report [38]. Probe sequences are listed in Supplemental Table S3.

### Transient genetic transformation of pear in vivo

The experimental procedures for in vivo transient genetic transformation were modified and adapted from previous studies [24, 39]. Virus-induced gene silencing was employed for gene silencing. Binary vectors pTRV2-PbMYB56 and 62sk-PbMYB56 were prepared and pooled into an infection suspension, following the same procedures as for the dual-luciferase assay. The suspension was applied to pear inflorescences as outlined in Supplemental Fig. S6A. Respective empty vectors served as controls. Treated inflorescences were bagged for 24 hours on the tree. Samples were collected at 3 DAA to assess transgene efficiency and at 7 DAA for morphological and cytological observations.

### FDA staining

Fluorescein diacetate (FDA) staining was used to evaluate cell activity. FDA, a fat-soluble, non-fluorescence substance, crosses cell membranes and is hydrolyzed by cellular esterase to produce a green fluorescent compound that cannot pass through cell membranes. Only active cells exhibit green fluorescence, while dead cells do not fluoresce. Pear fruitlets were sectioned and washed three times with phosphate-buffered saline (PBS, pH =8.0). Sections were incubated in 12.5 μg/mL FDA staining solution for 5 min, washed three times with distilled water (5 min each), and observed under a fluorescence microscope (M205FCA, Leica, Germany).

### Transgenic pear callus construction

Pear callus culture and subculture were performed as described previously [16]**.**

### Production of transgenic tomato lines and determination of parthenocarpic fruit set

The detailed procedures for generating transgenic tomato lines and determining parthenocarpic fruit set are outlined in a prior study [16]. Before flowering, the strongest and weakest flowers were removed from each bearing branch of transgenic tomato plants. The remaining flowers were emasculated to determine the parthenocarpic fruit set rate, with 3–4 emasculated flowers retained per bearing branch. Three branches were used as biological replicates.

### Y2H assays

The *PbDELLA* coding region was cloned into the PGBKT7 plasmid as a bait protein, and the *PbMYB56* coding sequence was cloned into the PGADT7 plasmid as a prey protein. Y2H assays were performed following a previous study [38].

### Pull-down assays

Recombinant proteins PbMYB56-His, PbDELLA-GST, and GST were produced via prokaryotic expression. Target proteins were purified using Ni-NTA Bind Resin for His-tagged proteins or GST-Resin for GST-tagged proteins. Detailed protocols were followed as described in a prior study [38].

### Luciferase complementation assay

Recombinant plasmids pCAMBIA1300-*PbMYB56-*Cluc and pCAMBIA1300-*PbDELLA*-Nluc were constructed. The luciferase complementation imaging assay was conducted following a previously established method [40]. Bioluminescence signal intensity was measured using a live plant imaging system (LumazonePylon2048B, Princeton, NJ, USA).

### Statistical analysis

Data were analyzed using SPSS software with one-way ANOVA (*P* < 0.06). Results are presented as means ± standard deviation (SD) of biological replicates.

## Supplementary Material

Web_Material_uhaf021

## Data Availability

All supporting data is included in the article or the supplementary files.
