# Supplementary material for: PbDELLA-PbMYB56-PbCYP78A6 module regulates GA4 + 7-induced pseudo-embryo development and parthenocarpy in pear (Pyrus bretschneideri)
Source: Hortic Res. 2025 Jan 21;12(5):uhaf021. doi: 10.1093/hr/uhaf021 (PMC11975393; doi:10.1093/hr/uhaf021)
Supplement: Web_Material_uhaf021 [file web_material_uhaf021.zip › Supplemental figure.docx]

SUPPLEMENTARY FIGURES


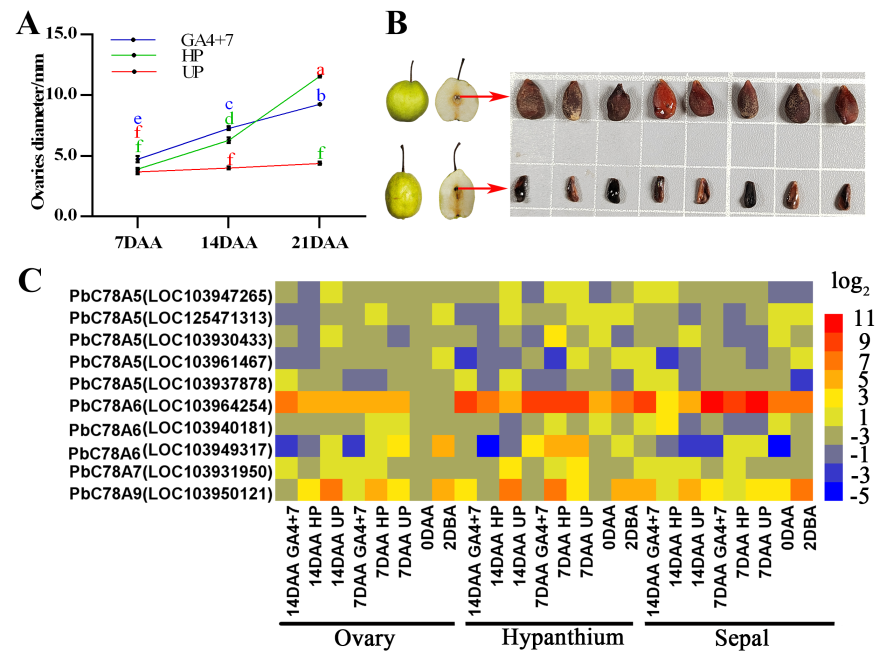


**Supplementary Figure S1.** Changes of treated ovaries diameter, seeds observation of HP and GA4+7 mature fruits, and CYP78A subfamily members’ expressions. (A) The diameter of UP, HP, and GA4+7 treated ovaries. (B), Seeded and seedless mature fruits and seeds produced by hand-pollination and GA4+7, respectively. (C), qRT-PCR was used to identified the relative expression of CYP78A subfamily members in UP, HP, and GA4+7 treated pear tissue including sepal, hypanthium and ovary derived from fruits. DAA, Days after anthesis; DBA, Days before anthesis.


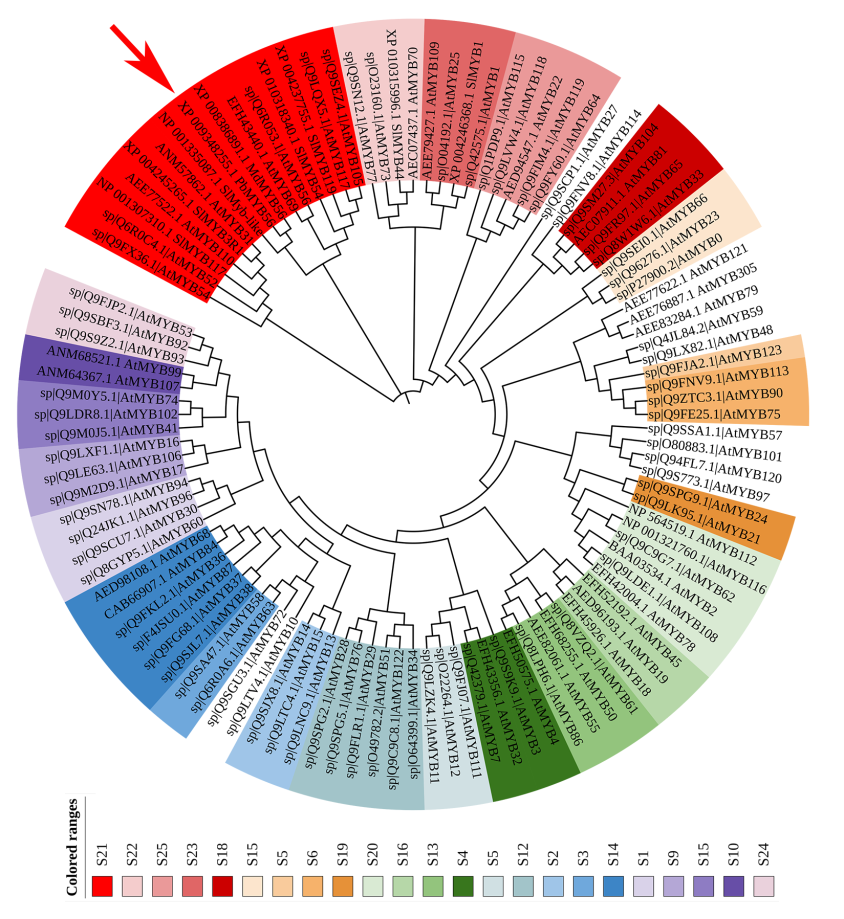


**Supplementary Figure S2.** Phylogenic analysis of PbMYB56 and representative members of different R2R3-MYB subgroups. The tree was constructed using the neighbor-joining method with 1000 bootstraps using putative amino acid full-length MYB sequences with MEGA X software.


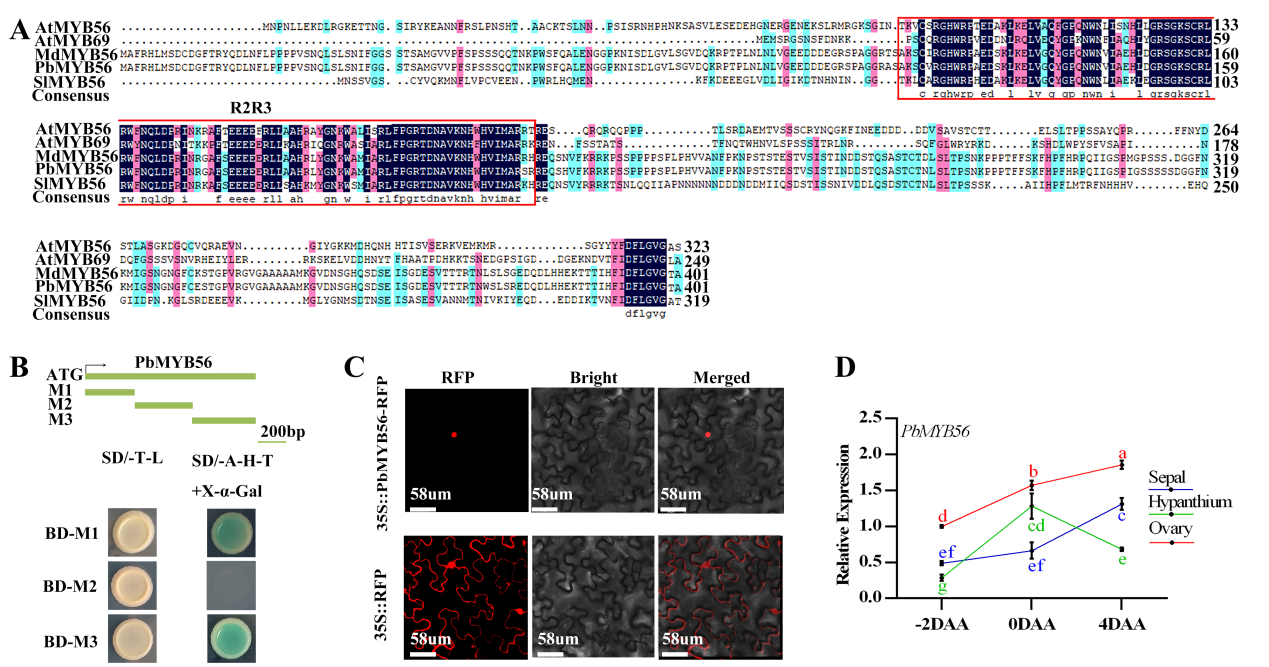


**Supplementary Figure S3.** Analysis of PbMYB56 protein sequences and its gene expression in response to pollination and fertilization. (A) Amino acid sequence alignment of PbMYB56 and orthologs from S21 subgroups. (B) Truncation of PbMYB56 and its self-activation in yeast. (C) Subcellular location of PbMYB56 in tobacco (N. *benthamiana*) lower epidermal cells. RFP: red fluorescent protein; bright field: visible light; merged: visible light merged with red fluorescence; 35S: RFP: empty pCAMBIA1300-RFP vector; 35S: PbMYB56-RFP: recombinant pCAMBIA1300- PbMYB56-RFP vector. Scale bars = 58 µm. (D) Relative expression levels of PbMYB56 in pear fruit-derived components, including the sepal, hypanthium, and ovary, at 2 days before anthesis (-2 DAA), 0 DAA, and 4 DAA. Three biological replicates were performed, and the error bars represent standard deviations. Statistical tests were performed by one-way analysis of variance followed by Tukey’s post-hoc test (P < 0.05). Different lowercase letters indicate significant differences at P < 0.05.


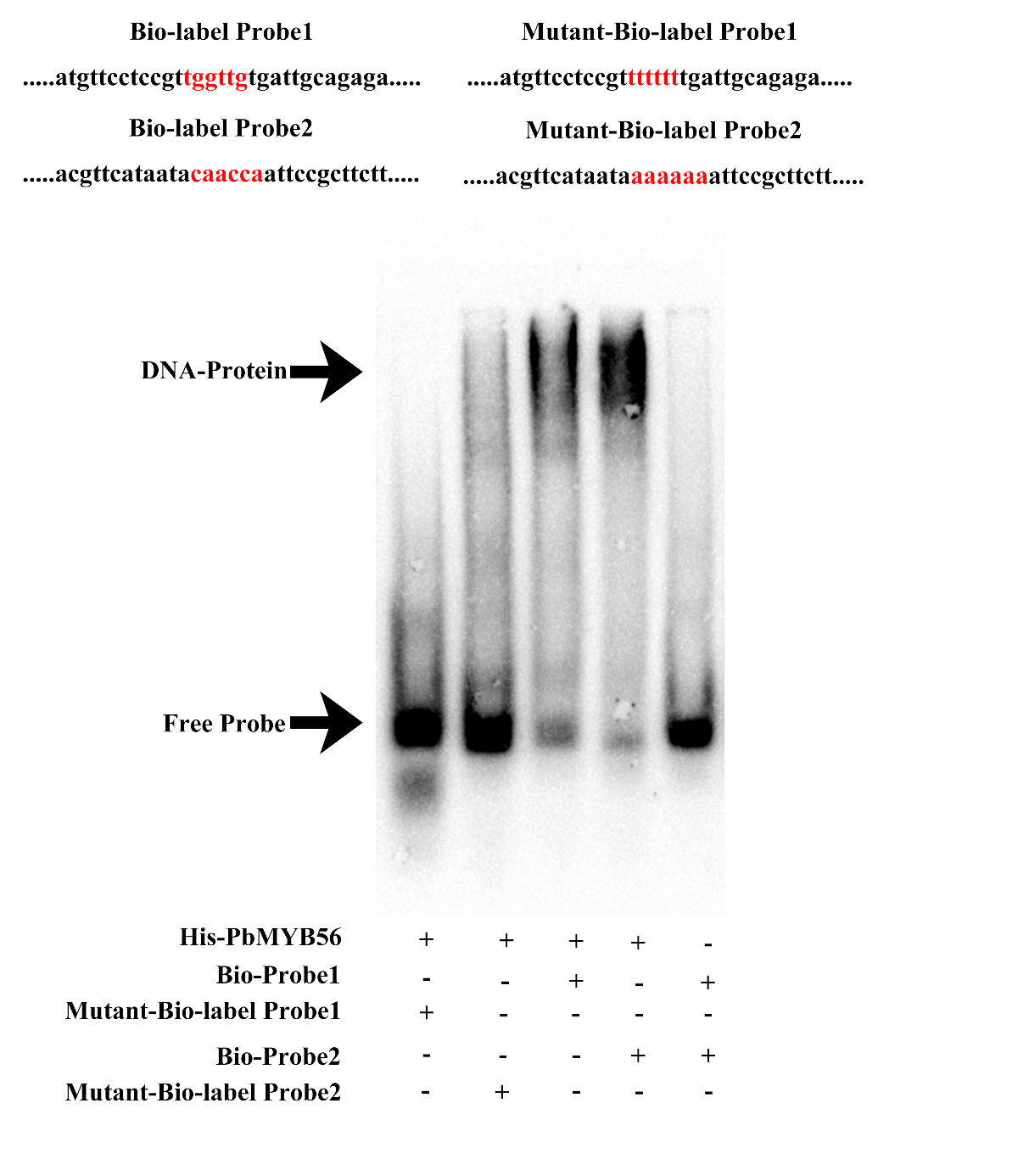


**Supplementary Figure S4.** PbMYB56 protein does not cause a gel-shift with mutant in the MYB-binding sites.

**
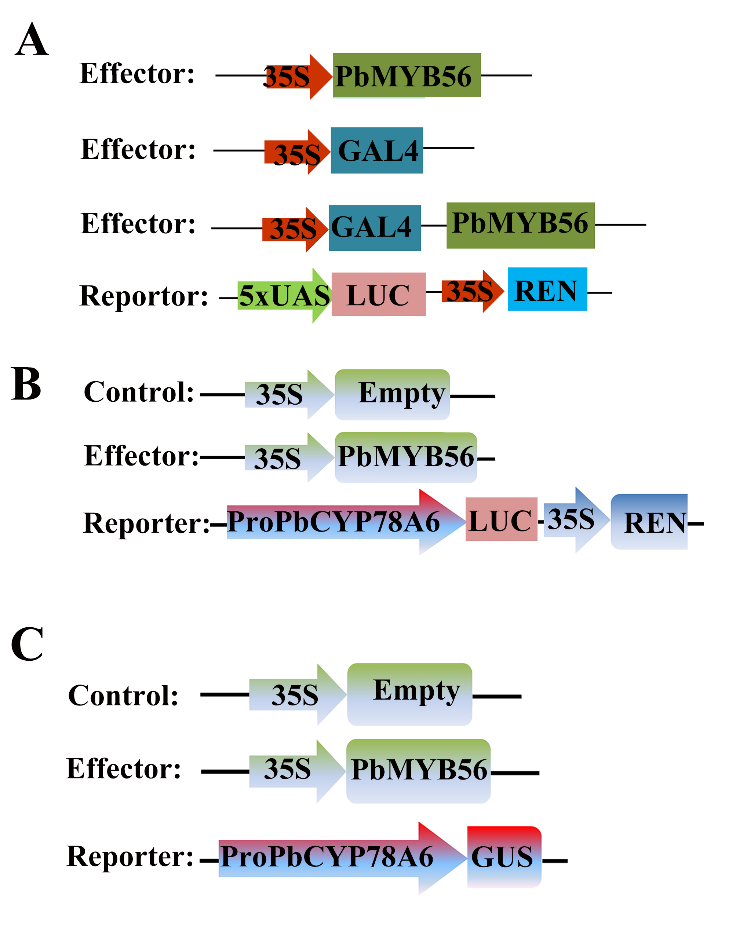
**

**Supplementary Figure S5.** A, the diagram of vectors construction in Figure 2C. B, the diagram of vectors construction in Figure 2E. C, the diagram of vectors construction in Figure 2G. pGreenII 62-SK was used for effector vector and pGreenII 0800 was used as reporter vector.


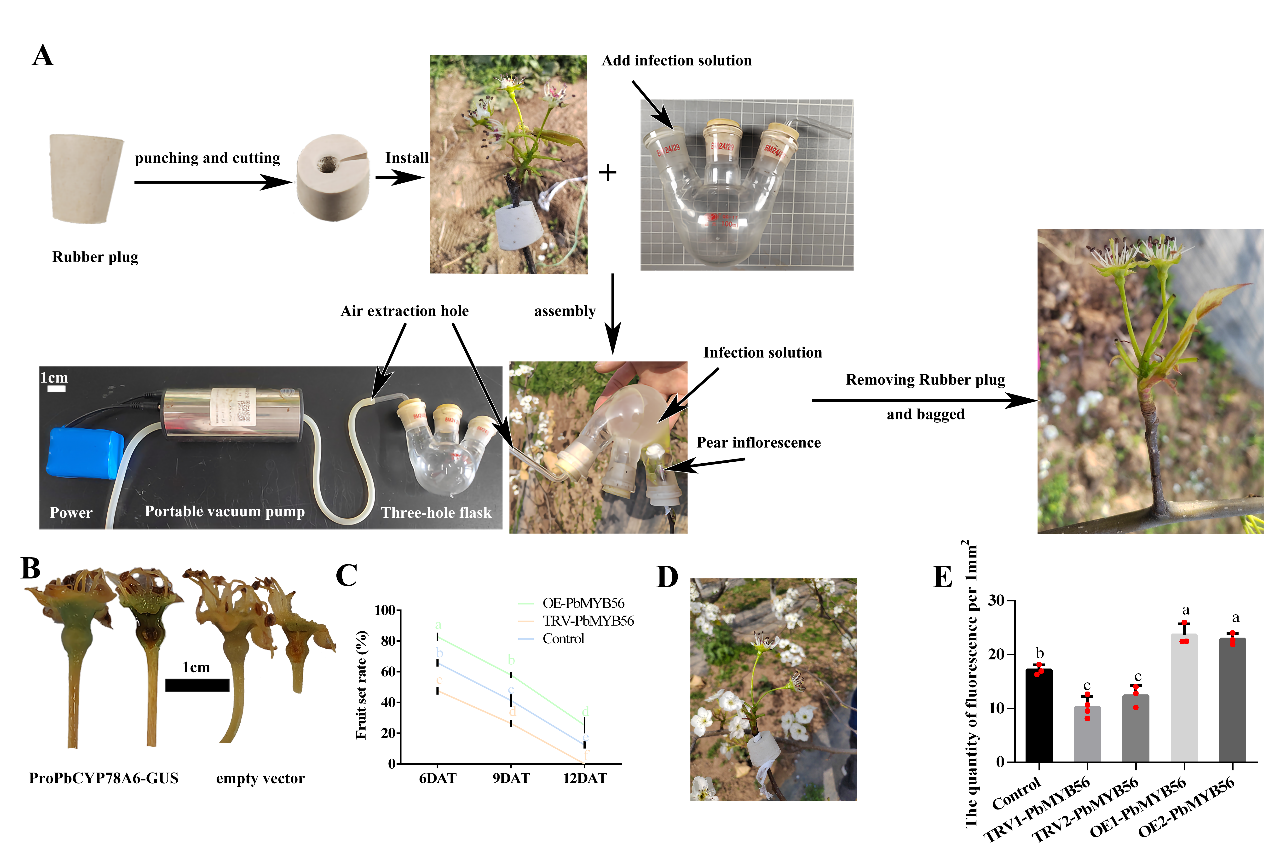


**Supplementary Figure S6.** Construction of a transient transgenic system in pear inflorescences on the tree in vivo and characterization of transgenes effects. (A) Process of establishing the in vivo transient transgenic system. (B) GUS staining to evaluate the practicability of the transgene method. (C) Fruit set rate of *PbMYB56*-TRV and *PbMYB56*-OE transient transgenic pear fruitlets. (D) the phenotype of *PbMYB56*-TRV2 after infection. (E) the quantity of fluorescence of *PbMYB56*-TRV and *PbMYB56*-OE transient transgenic pear fruitlets after FDA staining. Three biological replicates were performed, and the error bars represent standard deviations. Statistical tests were performed by one-way analysis of variance followed by Tukey’s post-hoc test (*P < 0.05, ***P < 0.001). Each biological replicate contains 6 fruitlets.


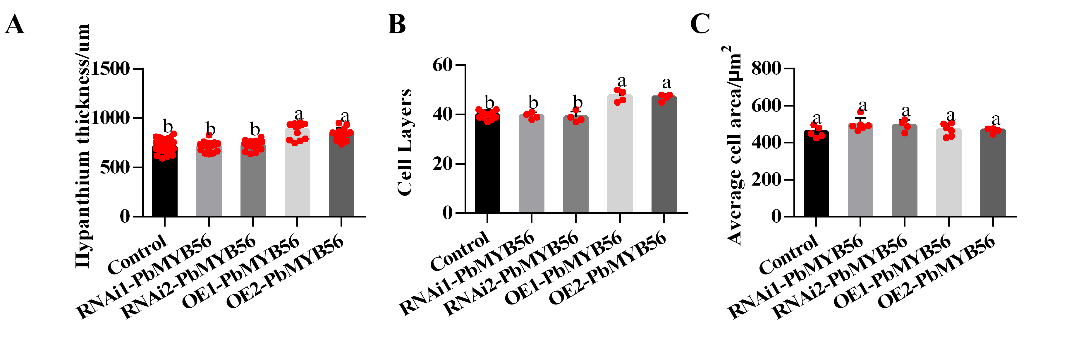


**Supplementary Figure S7.** Quantification of paraffin-embedded ovary sections with transient overexpression and RNA interference of *PbMYB56* in pear. (A) Hypanthium thickness, (B) cell layers and (C) average cell area in ovaries with transient overexpression and RNA interference of PbMYB56 in pear.


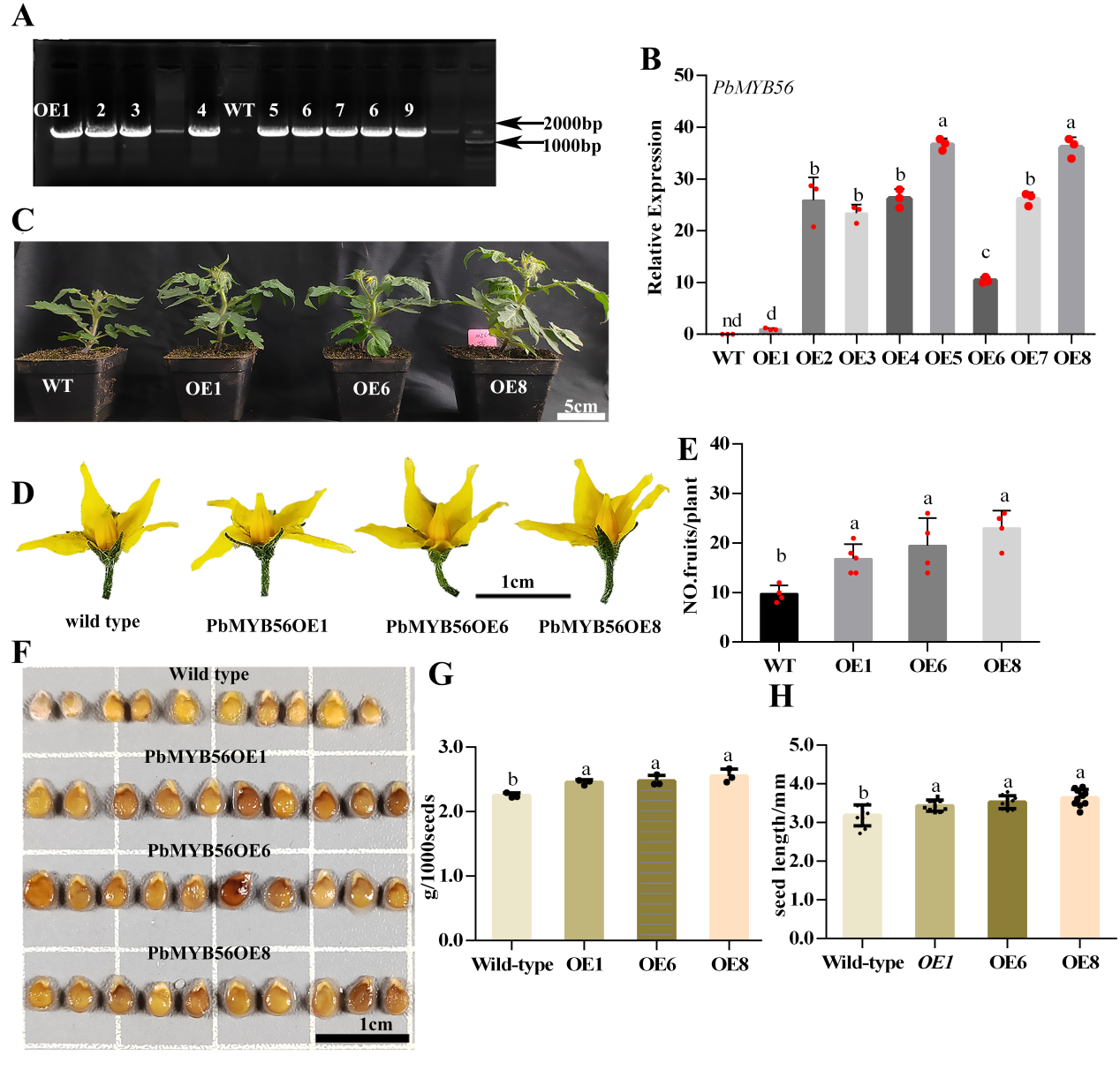


**Supplementary Figure S8.** Characterization of transgenic tomato lines with ectopic overexpression (OE) of *PbMYB56*. (A) DNA detection of *PbMYB56* in transgenic tomato plants by polymerase chain reaction (PCR). (B) Reverse transcription-quantitative PCR identification of *PbMYB56*-OE in eight transgenic lines compared with the non-transgenic control. *SlACTIN* was used for internal control amplification. (C) Phenotypes of *PbMYB56*-OE lines at the end of vegetative growth. (D) Flower phenotypes of *PbMYB56*-OE lines. (E) Number of fruits produced by *PbMYB56*-OE lines. At least 4 plants were used for analysis. (F) Seed phenotypes of *PbMYB56*-OE lines. (G) 1000-seeds weight of *PbMYB56*-OE lines. (H) Length of seeds produced by *PbMYB56*-OE lines. Significant differences as determined by one-way analysis of variance (P < 0.05) are indicated using different lowercase letters (a, b, c, d). Data represent the means (±standard deviation) of three biological replicates (n = 3 in B and E; n > 9 in G and H).


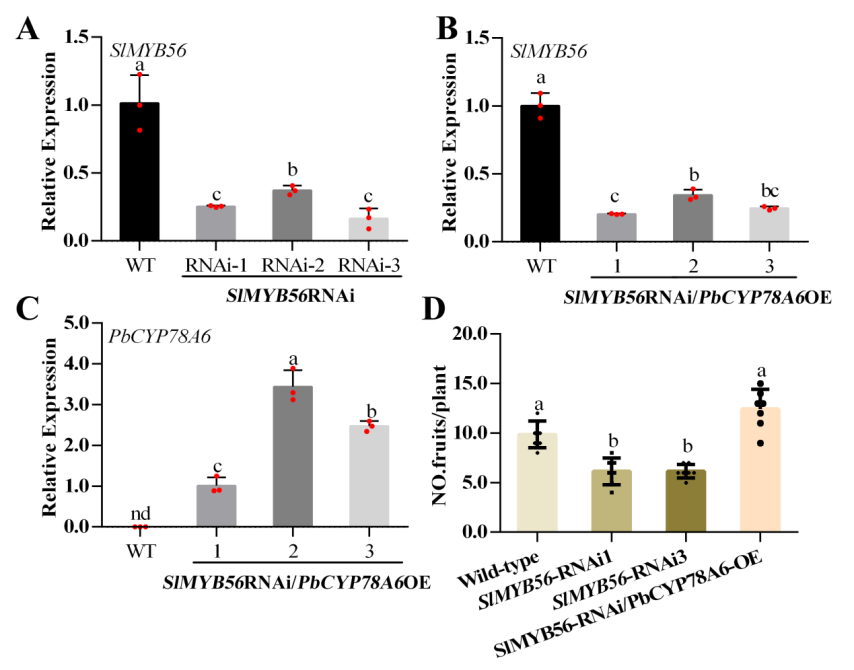


**Supplementary Figure S9.** Characterization of *SlMYB56*-RNA interference (RNAi) and *SlMYB56*-RNAi/*PbCYP78A6*-overexpression (OE) tomato lines. (A) Reverse transcription-quantitative polymerase chain reaction identification of *SlMYB56*- silenced transgenic lines. (B) Relative expression of *SlMYB56* in *SlMYB56*-RNAi/*PbCYP78A6*-OE tomato lines. (C) Relative expression of *PbCYP78A6* in *SlMYB56*-RNAi/*PbCYP78A6*-OE tomato lines. (D) The number of fruits produced by transgenic lines. Significant differences as determined by one-way analysis of variance (P < 0.05) are indicated using different lowercase letters (a, b, c, d). Data represent the means (±standard deviation) of three biological replicates (n = 3 in A-C; n =4 in D).


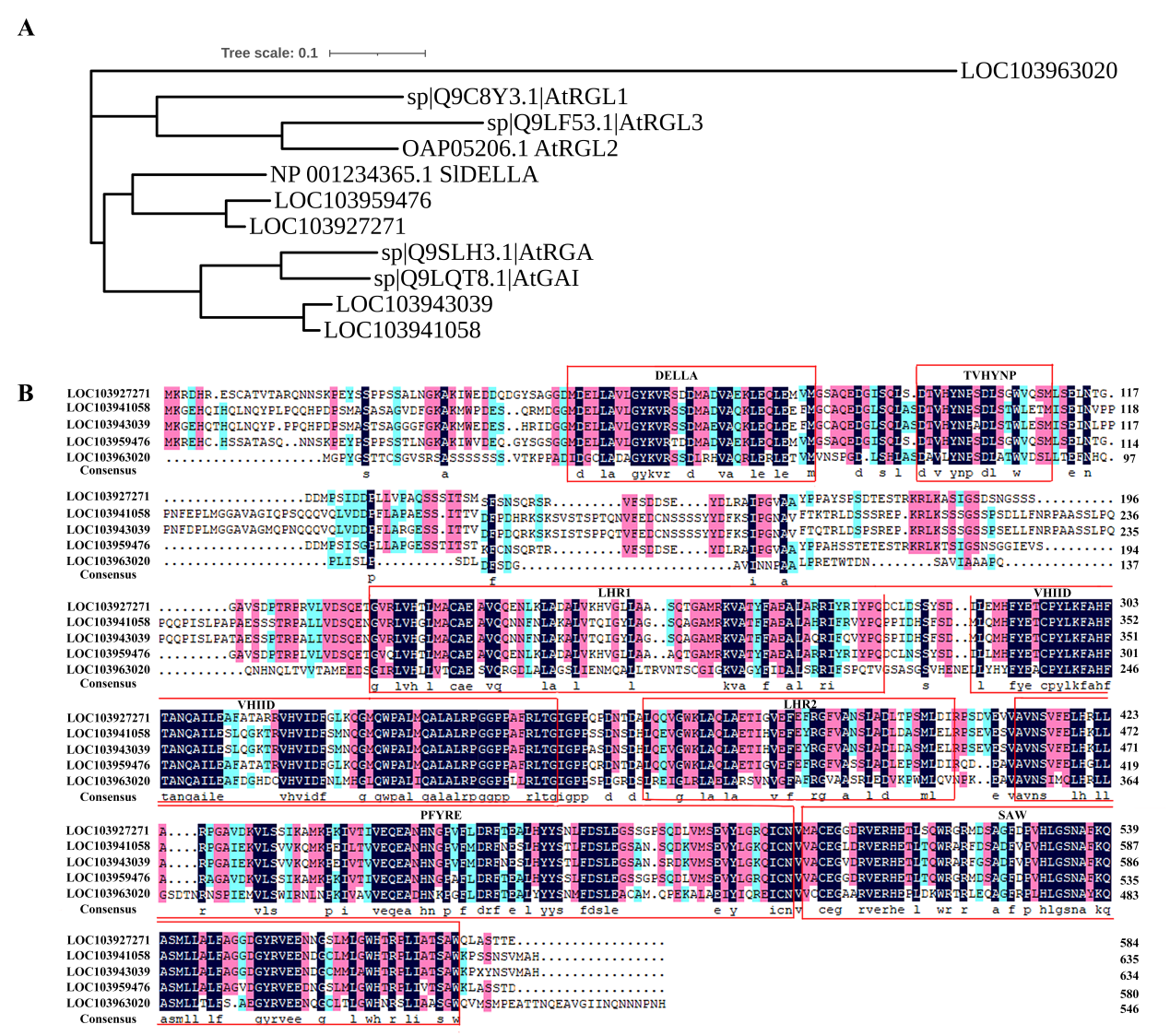


**Supplementary Figure S10.** Characterization of DELLA proteins in pear. A, Phylogenic analysis of PbDELLA proteins in pear and DELLA proteins (AtRGA, AtRGL1, AtRGL2, AtRGL3) in Arabidopsis and SlDELLA in tomato. The tree was constructed using the neighbor-joining method with 1000 bootstraps using putative amino acid full-length MYB sequences with MEGA X software. B, Five DELLA proteins sequences were retrieved from the pear genome based on the BLAST function. DELLA, TVHYNP, LHR1, VHIID, LHR2, PFYRE, and SAW domains are framed by red lines.


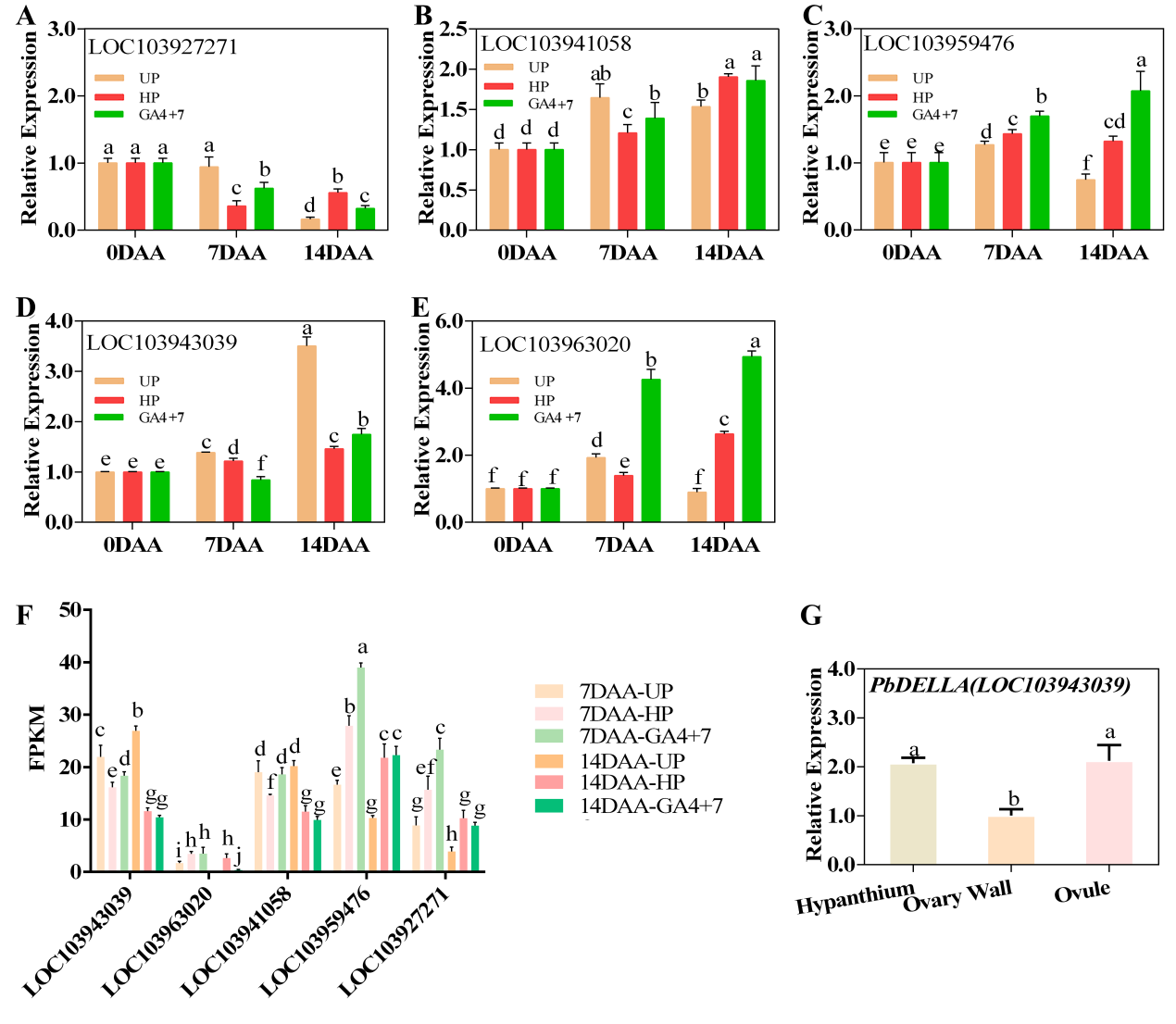


**Supplementary Figure S11.** Screening key *PbDELLA* gene related to fruit set in pear. (A-E) Relative expression of five *PbDELLA* genes at 0, 7, and 14 days after anthesis (DAA) following GA4+7 and HP induced fruit set. (F) The FPKM value of *PbDELLA* genes in pear. (G) Relative expression of PbDELLA gene (LOC103943039) in pear fruit tissue including hypanthium, ovary wall and ovule. Different lowercase letters indicate statistically significant differences as determined by one-way analysis of variance (P < 0.05). Data represent the means (±standard deviation) of three biological replicates (n = 9).


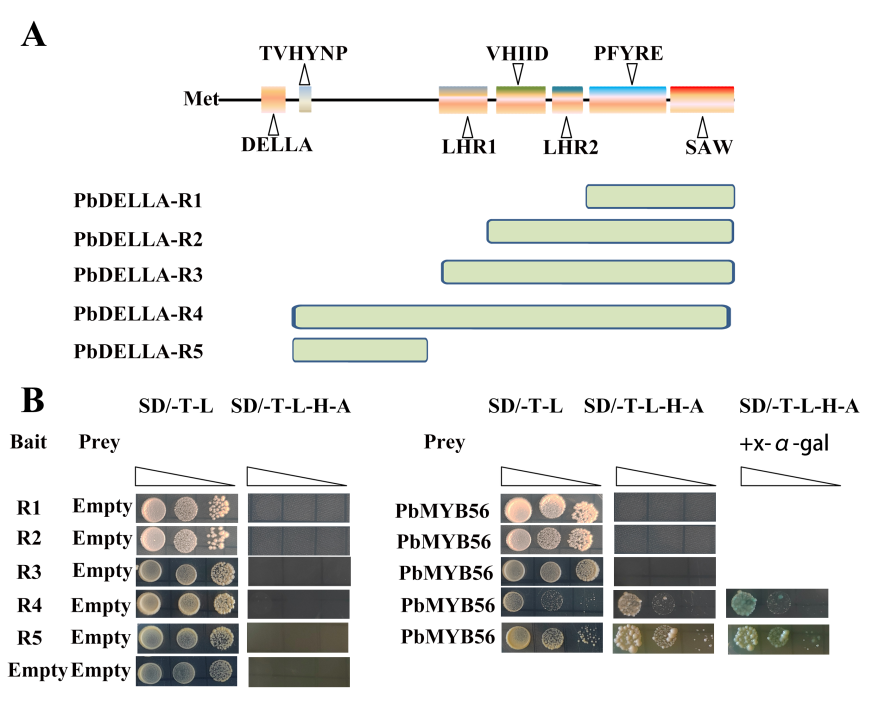


**Supplementary Figure S12.** PbMYB56 interacts with PbDELLA through the specific protein segment containing TVHYNP. (A) Diagram of truncated PbDELLA protein. (B) PbDELLA protein segments used in the yeast-two-hybrid assay. (C) Specific PbDELLA segment interacting with PbMYB56.


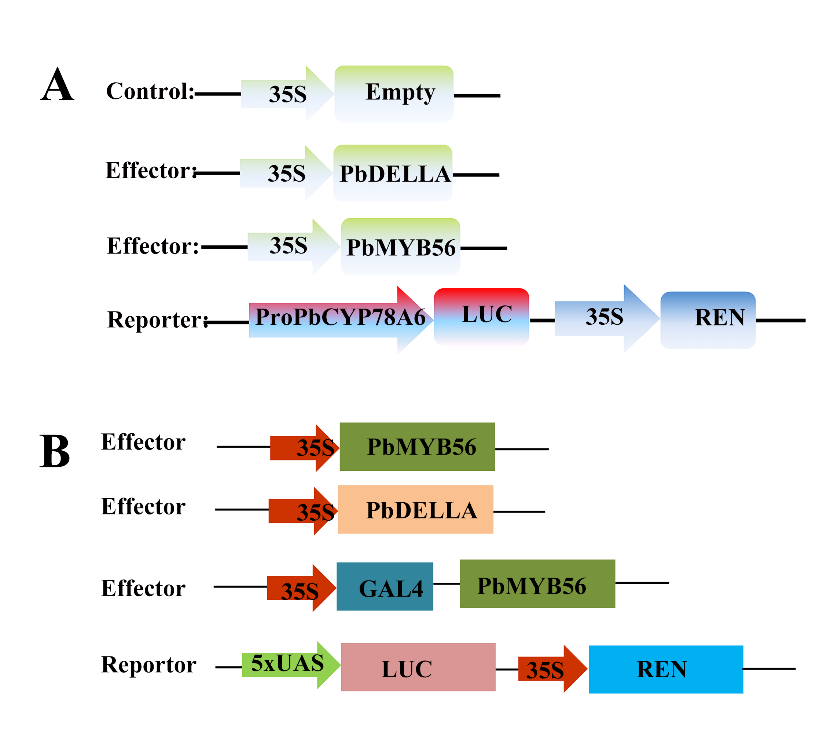


**Supplementary Figure S13.** A, the diagram of vectors construction in Figure 5J-H. B, the diagram of vectors construction in Figure 5I-K. pGreenII 62-SK was used for effector vector and pGreenII 0800 was used as reporter vector.


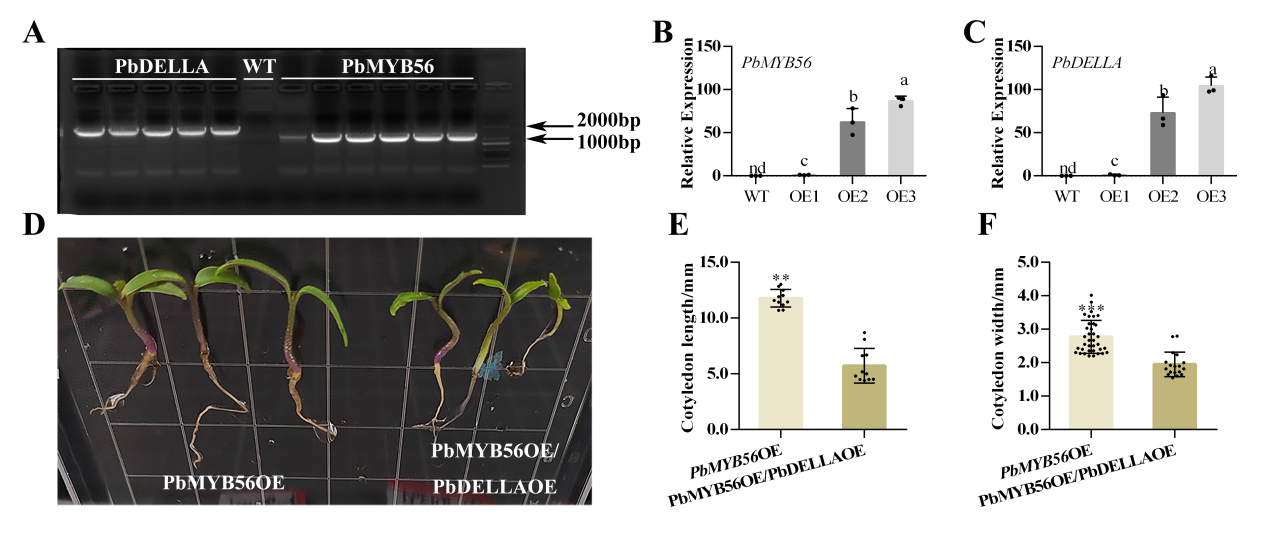


**Supplementary Figure S14.** Characterization of transgenic tomato lines with ectopic overexpression (OE) of *PbMYB56* and *PbDELLA*. (A) DNA detection of *PbMYB56* and *PbDELLA* in *PbMYB56*-OE/*PbDELLA*-OE transgenic tomato plants by polymerase chain reaction (PCR). (B) Reverse transcription-quantitative PCR identification of *PbMYB56* in *PbMYB56*-OE/*PbDELLA*-OE transgenic lines compared with the non-transgenic control. *SlACTIN* was used for internal control amplification. (C) Relative expression of *PbDELLA* in *PbMYB56*-OE/*PbDELLA*-OE transgenic lines. (D) Phenotype of transgenic seedlings germinated from seeds produced by *PbMYB56*-OE, and *PbMYB56*-OE/*PbDELLA*-OE transgenic lines. (E) Tomato cotyledon length in (D). F, Tomato cotyledon width in D.
