# Supplementary material for: PbDELLA-PbMYB56-PbCYP78A6 module regulates GA4 + 7-induced pseudo-embryo development and parthenocarpy in pear (Pyrus bretschneideri)
Source: Hortic Res. 2025 Jan 21;12(5):uhaf021. doi: 10.1093/hr/uhaf021 (PMC11975393; doi:10.1093/hr/uhaf021)
Supplement: Web_Material_uhaf021 [file web_material_uhaf021.zip › Supplemental tables.docx]

**SUPPLEMENTARY TABLES**

| **Supplementary Table S1 List of primers for qRT-PCR** | |
| --- | --- |
| **Primers** | **Sequences (5’-3’)** |
| q-*PbMYB56*(LOC103939879)-F | AGTCTGCCTCAACATGCACT |
| q-*PbMYB56*(LOC103939879)-R | GATCCAATGATTTGGGGGCG |
| q-Sl*MYB56*-F | TCAGCATCTGAGTCAGTAGCCA |
| q-Sl*MYB56*-R | GTGGCTCCAACTCCAAGAAAA |
| q-PbCYP78A6(LOC103964254)-F | ATTTCGCGGGATCCTGAAGT |
| q-PbCYP78A6(LOC103964254)-R | GGTGCAAGCCTAAGATCCGA |
| q-*PbDELLA(LOC103963020)-*F | ATGCGTATAAGCAGGCGAGT |
| q-*PbDELLA(LOC103963020)*-R | CCGAAGCCGCGATAAGAGAA |
| q-*PbDELLA(LOC103943039)*-F | CCCAGCCCTCATTGTCGATT |
| q-*PbDELLA(LOC103943039)*-R | AGAGCTTTGGCGAGGTTGAA |
| q-*PbDELLA(LOC103927271)*-F | CGACATCCGCCCGTCAG |
| q-*PbDELLA(LOC103927271)*-R | CTCCTGCTCCACAATTGTCAC |
| q-*PbDELLA(LOC103959476)*-F | ATTCCGGCGGGATTGAAGTC |
| q-*PbDELLA(LOC103959476)*-R | TGAGTGTGTGGACGAGTTGG |
| q-*PbDELLA (LOC103941058)*-F | AGTATGCTGCTGGCGTTGTT |
| q-*PbDELLA (LOC103941058)*-R | TTGGAAGATGGCTTCCACGC |
| q-*PbActin-7*-F | CCATCCAGGCTGTTCTCTC |
| q-*PbActin-7*-R | GCAAGGTCCAGACGAAGG |
| q-PbCYP78A5(LOC103930433)-F | CGAGACAGTATCGGGTGCAA |
| q-PbCYP78A5(LOC103930433)-R | AGCTCCTCCCAAACACACTC |
| q-PbCYP78A5(LOC103930433)-F | CTTCAACAGAGCGATGGGGT |
| q-PbCYP78A5(LOC103930433)-R | AACCTCAGAGCCAGCGATTC |
| q-PbCYP78A5(LOC103961467)-F | CAAGTGGTTACCGGCAAAGC |
| q-PbCYP78A5(LOC103961467)-R | CTCGTAATGCACTGCGTGGG |
| q-PbCYP78A5(LOC103947265)-F | CTTCAACAGAGCGATGGGGT |
| q-PbCYP78A5(LOC103947265)-R | AACCTCAGAGCCAGCGATTC |
| q-PbCYP78A5(LOC103937878)-F | ACGGCGATTACAAATCGAAGAG |
| q-PbCYP78A5(LOC103937878)-R | CAAGAAACCCAAGTGGTGGG |
| q-PbCYP78A5(LOC125471313)-F | TATGCCCGGGTAAAGCGTTG |
| q-PbCYP78A5(LOC125471313)-R | GAAAGGTCAACAGACTCCGC |
| q-PbCYP78A6(LOC103949317)-F | GCCAAGCCTTATCCTCCACA |
| q-PbCYP78A6(LOC103949317)-R | ATATTTCCCCACGCTGGACC |
| q-PbCYP78A9(LOC103950121)-F | CAGGAGTTTCGGCCTGAGAG |
| q-PbCYP78A9(LOC103950121)-R | TCATCCCCAATGTCTTCCCG |
| q-PbCYP78A6(LOC103940181)-F | CCCACCAGGCCCACTTTTAT |
| q-PbCYP78A6(LOC103940181)-R | CTAGTGGGTCCGGCCAAAAC |

| **Supplementary Table S2 List of cloning primers with adaptors for designated vectors** | | | | |
| --- | --- | --- | --- | --- |
| **Primers** | | | **Sequences (5’-3’)** | |
| 62SK-PbMYB56-F | | TAGAACTAGTGGATCCATGGCGTTTCGACATCTGAT | | |
| 62SK-*PbMYB56*-R | | CGGTATCGATAAGCTTAGCAGTGCCTACTCCAAGGAAA | | |
| AD-PbMYB56-F | | GCCATGGAGGCCAGTGAATTCATGGCGTTTCGACATCTGAT | | |
| AD-*PbMYB56*-R | | CAGCTCGAGCTCGATGGATCCAGCAGTGCCTACTCCAAGGAAA | | |
| 32a-PbMYB56-F | | TATCGGATCCGAATTCATGGCGTTTCGACATCTGAT | | |
| 32a-*PbMYB56*-R | | GTGCGGCCGCAAGCTTAGCAGTGCCTACTCCAAGGAAA | | |
| 0800-Pro*PbCYP78A6*-F | | CGGTATCGATAAGCTTGTAATTTCCAGCTTCAAAACAAAAGTT | | |
| 0800-Pro*PbCYP78A6*-R | | TAGAACTAGTGGATCCGGCGGAATTGTAGATTGTAGAGAGAG | | |
| 1301-Pro*PbCYP78A6*-F | | CCATGATTACGAATTCGTAATTTCCAGCTTCAAAACAAAAGTT | | |
| 1301-Pro*PbCYP78A6*-R | | CTCAGATCTACCATGGGGCGGAATTGTAGATTGTAGAGAGAG | | |
| Abai-Pro*PbCYP78A6*-F | | AAAAGCTTGAATTCGAGCTCAGTGATTCCTTACAACTTGTTCAGT | | |
| Abai-Pro*PbCYP78A6*-R | | ATGCCTCGAGGTCGACTAACCTACTTAGTGAGTCTTACCGG | | |
| BD-PbMYB56-F | | ATGGCCATGGAGGCCGAATTCATGGCGTTTCGACATCTGAT | | |
| BD-*PbMYB56*-R | | CCGCTGCAGGTCGACGGATCCAGCAGTGCCTACTCCAAGGAAA | | |
| BD1-PbMYB56-F | | ATGGCCATGGAGGCCGAATTCATGGCGTTTCGACATCTGAT | | |
| BD1-*PbMYB56*-R | | CCGCTGCAGGTCGACGGATCCAGCACTAGTTCTCCCACCAGC | | |
| BD2-PbMYB56-F | | ATGGCCATGGAGGCCGAATTCGCTGGTGGGAGAACTAGTGCT | | |
| BD2-*PbMYB56*-R | | CCGCTGCAGGTCGACGGATCCCTCTTGAAAACATGGGATTGCTC | | |
| BD3-PbMYB56-F | | ATGGCCATGGAGGCCGAATTCGAGCAATCCCATGTTTTCAAGAG | | |
| BD3-*PbMYB56*-R | | CCGCTGCAGGTCGACGGATCCAGCAGTGCCTACTCCAAGGAAA | | |
| 1300-PbMYB56-F | | CCGGGGATCCTCTAGAATGGCGTTTCGACATCTGAT | | |
| 1300-*PbMYB56*-R | | AGGAGGCCATGTCGACAGCAGTGCCTACTCCAAGGAAA | | |
| TRV-PbMYB56-F | | GCCTCCATGGGGATCTGTCAGAGGCCATTGGAGACCA | | |
| TRV-*PbMYB56*-R | | ATGCCCGGGCCTCGAGCCATGATCACATGCCAATGGT | | |
| OE-PbMYB56-F | | GGGGACAAGTTTGTACAAAAAAGCAGGCTGCATGGCGTTTCGACATCTGAT | | |
| OE-*PbMYB56*-R | | GGGGACCACTTTGTACAAGAAAGCTGGGTC AGCAGTGCCTACTCCAAGGAAA | | |
| RNAi-SlMYB56-F | | GGGGACAAGTTTGTACAAAAAAGCAGGCTGCGCACTAATCTTTCACTCACTCCATC | | |
| RNAi-Sl*MYB56*-R | | GGGGACCACTTTGTACAAGAAAGCTGGGTC TATTGGTCATGTTGTTGGCTACTG | | |
| BD-PbDELLA-F | | ATGGCCATGGAGGCCGAATTCATGAAAGGGGAGCACCAGACTC | | |
| BD-PbDELLA-R | | CCGCTGCAGGTCGACGGATCCGTGAGCCATGACCGAGTTATAAGC | | |
| BD-PbDELLA-F-F | | ATGGCCATGGAGGCCGAATTCATGAAAGGGGAGCACCAGACTC | | |
| BD-PbDELLA-F1-R | | CCGCTGCAGGTCGACGGATCCGTCAGAAGCGAGCTGAGAGAGTC | | |
| BD-PbDELLA-F2-R | | CCGCTGCAGGTCGACGGATCCTTCCTGCGAATCGACAATGA | | |
| BD-PbDELLA-F3-R | | CCGCTGCAGGTCGACGGATCCGGAGTGGTCGATCGGCG | | |
| BD-PbDELLA-R1-F | | ATGGCCATGGAGGCCGAATTCGAGTCGGTGGCGGTCAACT | | |
| BD-PbDELLA-R2-F | | ATGGCCATGGAGGCCGAATTCAACTCCGACCATCTGCAGGA | | |
| BD-PbDELLA-R3-F | | ATGGCCATGGAGGCCGAATTCTTCTCAGACATGCTACAGATGCAC | | |
| BD-PbDELLA-R4-F | | ATGGCCATGGAGGCCGAATTCGATTCGCAGGAAAACGGAGTC | | |
| BD-PbDELLA-R-R | | CCGCTGCAGGTCGACGGATCCGTGAGCCATGACCGAGTTATAAGC | | |
| BD-PbDELLA-R5-F | | ATGGCCATGGAGGCCGAATTCGGACTCTCTCAGCTCGCTTCTGA | | |
| BD-PbDELLA-R5-R | | CCGCTGCAGGTCGACGGATCCTTCCTGCGAATCGACAATGA | | |
| 4T-1-PbDELLA-F | | GGTTCCGCGTGGATCCATGAAAGGGGAGCACCAGACTC | | |
| 4T-1-PbDELLA-R | | GGCCGCTCGAGTCGACGTGAGCCATGACCGAGTTATAAGC | | |
| nLUC-PbDELLA-F | | TCGGTACCCGGGATCCATGAAAGGGGAGCACCAGACTC | | |
| nCLU-PbDELLA-R | | ACGAGATCTGGTCGAC GTGAGCCATGACCGAGTTATAAGC | | |
| cLUC-PbMYB56-F | | ACGCGTCCCGGGGCGGTACC ATGGCGTTTCGACATCTGAT | | |
| cLUC-*PbMYB56*-R | | TACGAACGAAAGCTCTGCAGAGCAGTGCCTACTCCAAGGAAA | | |
| 203-Link-1300-F | | TCGAGGCTCAGCAGGGTACCAATTCGGTCCCCAGATTAGCC | | |
| 203-Link-1300-R | | CATGTATAATTCGCGGTACCGGCGCCGGTGGAGTG | | |
| 1300-PbDELLA-F | | CCGGGGATCCTCTAGAATGAAAGGGGAGCACCAGACT | | |
| 1300-*PbDELLA*-R | | AGGAGGCCATGTCGACGTGAGCCATGACCGAGTTATAAGC | | |
| 62SK-PbDELLA-F | | TAGAACTAGTGGATCCATGAAAGGGGAGCACCAGACTC | | |
| 62SK-PbDELLA-R | | CGGTATCGATAAGCTTGTGAGCCATGACCGAGTTATAAGC | | |
| 62SK-GAL4-F | | TAGAACTAGTGGATCCATGAAGCTACTGTCTTCTATCGAACAAGC | | |
| 62SK-GAL4-R | | CGGTATCGATAAGCTTCGATACAGTCAACTGTCTTTGACCTTTG | | |
| 62SK-GAL4-PbMYB56-F | | TAGAACTAGTGGATCCATGAAGCTACTGTCTTCTATCGAACAAGC | | |
| 62SK-GAL4-PbMYB56-R | | | CGGTATCGATAAGCTTAGCAGTGCCTACTCCAAGGAAA | |
| **Supplementary Table S3 List of probes designed for the promoter of *PbCYP78A6* containing MBSs** | | | |  |
| **Primers** | | | **Sequences (5’-3’)** |  |
| proPbCYP78A6-MBS1 | | | atgttcctccgttggttgtgattgcagaga |  |
| proPbCYP78A6-MBS1 | | | tctctgcaatcacaaccaacggaggaacat |  |
| proPbCYP78A6-MBS2 | | | acgttcataatacaaccaattccgcttctt |  |
| proPbCYP78A6-MBS2  proPbCYP78A6-MBS1-Mutant  proPbCYP78A6-MBS1- Mutant  proPbCYP78A6-MBS2-Mutant  proPbCYP78A6-MBS2-Mutant | | | aaagaagcggaattggttgtattatgaacgt  atgttcctccgttttttttgattgcagaga  tctctgcaatcaaaaaaaacggaggaacat  acgttcataataaaaaaaattccgcttctt  aagaagcggaattttttttattatgaacgt |  |
